# Boosting the Potential of Chemotherapy in Advanced Breast Cancer Lung Metastasis via Micro‐Combinatorial Hydrogel Particles

**DOI:** 10.1002/advs.202205223

**Published:** 2023-01-22

**Authors:** Anna Lisa Palange, Daniele Di Mascolo, Miguel Ferreira, Peter J. Gawne, Raffaele Spanò, Alessia Felici, Luca Bono, Thomas Lee Moore, Marco Salerno, Andrea Armirotti, Paolo Decuzzi

**Affiliations:** ^1^ Laboratory of Nanotechnology for Precision Medicine Fondazione Istituto Italiano di Tecnologia Via Morego 30 Genoa 16163 Italy; ^2^ Analytical Chemistry Facility Fondazione Istituto Italiano di Tecnologia Via Morego 30 Genoa 16163 Italy; ^3^ Materials Characterization Facility Fondazione Istituto Italiano di Tecnologia Via Morego 30 Genoa 16163 Italy; ^4^ Present address: Harvard Medical School, Department of Radiology Massachusetts General Hospital Boston MA 02114 USA; ^5^ Present address: Division of Oncology, Department of Medicine and Department of Pathology Stanford University School of Medicine Stanford CA 94305 USA

**Keywords:** docetaxel, drug delivery, hydrogels, pulmonary metastasis, soft lithography, theranostics

## Abstract

Breast cancer cell colonization of the lungs is associated with a dismal prognosis as the distributed nature of the disease and poor permeability of the metastatic foci challenge the therapeutic efficacy of small molecules, antibodies, and nanomedicines. Taking advantage of the unique physiology of the pulmonary circulation, here, micro‐combinatorial hydrogel particles (µCGP) are realized via soft lithographic techniques to enhance the specific delivery of a cocktail of cytotoxic nanoparticles to metastatic foci. By cross‐linking short poly(ethylene glycol) (PEG) chains with erodible linkers within a shape‐defining template, a deformable and biodegradable polymeric skeleton is realized and loaded with a variety of therapeutic and imaging agents, including docetaxel‐nanoparticles. In a model of advanced breast cancer lung metastasis, µCGP amplified the colocalization of docetaxel‐nanoparticles with pulmonary metastatic foci, prolonged the retention of chemotoxic molecules at the diseased site, suppressed lesion growth, and boosted survival beyond 20 weeks post nodule engraftment. The flexible design and modular architecture of µCGP would allow the efficient deployment of complex combination therapies in other vascular districts too, possibly addressing metastatic diseases of different origins.

## Graphical abstract

Micro‐Combinatorial hydroGel Particles (µCGP) with a natural tropism for the pulmonary circulation are realized via soft lithography to amplify the deposition of cytotoxic agents in cancer metastasis. In advanced triple negative breast cancer lung metastasis models, µCGP carrying docetaxel nanomedicines boosted the potential of chemotherapy extending survival beyond 5 months.

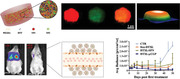

## 1 Introduction

Current clinical standards for the treatment of breast cancer, including surgical resection, radio‐chemotherapy, targeted and immuno‐therapy, are successfully addressing the disease in millions of women, returning a 5‐year survival rate close to 100%.^[^ ^1^, ^2^, ^3^, ^4^ ^]^ This statement is indeed true for a “local disease” diagnosis, whereby malignant cells are either confined within the tissue of origin or have moderately spread in the surrounding area. Unfortunately, this picture dramatically changes for a diagnosis of “metastatic disease”, for which breast cancer cells have colonized distant organs and lymph nodes originating multiple malignant foci.^[^ ^2^, ^5^, ^6^ ^]^ Under this condition, surgical intervention is only limited to the removal of sufficiently large and accessible malignant masses,^[^ ^7^, ^8^ ^]^ thus turning systemic therapy into the mainstay option. However, the periodic administration of cytotoxic molecules, including platinum‐based drugs, doxorubicin, and taxanes; small inhibitors, and antibodies is hindered by their inefficient accumulation at the malignant foci that inevitably results in systemic toxicity.^[^ ^9^ ^]^ Therefore, it is not surprising that metastatic breast cancer is associated with a dismal prognosis and a 5‐year survival rate of only 30%.^[^ ^1^ ^]^ This figure is further aggravated in triple negative breast cancer (TNBC) – which is insensitive to targeted therapies, extremely aggressive, and prone to colonize the lungs^[^ ^6^, ^10^ ^]^ – where the survival rate at 5 years plunges below 10%, making this essentially an uncurable disease.^[^ ^1^ ^]^

Nanomedicine has provided a much‐needed boost toward improving the potency of virtually any anti‐cancer drug, mostly by optimizing bioavailability and tissue‐specific deposition while limiting off‐site effects.^[^ ^11^, ^12^ ^]^ In treating primary breast tumors, nanomedicines have been engineered following the dogma of the enhanced permeability and retention (EPR) effect reciting that systemically administered nanoparticles would accumulate intratumorally upon passive extravasation across the hyperpermeable malignant blood vessels and be retained thereof by the dense extracellular matrix and poor lymphatic drainage.^[^ ^13^ ^]^ Therefore, micelles, liposomes, dendrimers, polymeric and metallic nanoparticles, and combination thereof have been all designed to exhibit a characteristic size smaller than 200 nm and a spheroidal shape. Although some of these nanomedicines have been also approved for the treatment of metastatic breast cancer, such as doxorubicin‐loaded liposomes (Doxil) and paclitaxel‐albumin complexes (Abraxane),^[^ ^14^, ^15^, ^16^ ^]^ the EPR effect is essentially non‐existent in small and poorly vascularized metastatic nodules, where compression of the fast expanding malignant tissue would even impair the infiltration of immune cells.^[^ ^17^, ^18^, ^19^ ^]^

To circumvent this delivery limitation, nanoparticles have been designed to simultaneously recognize multiple receptors expressed on the membrane of the breast cancer cells and malignant vasculature.^[^ ^20^, ^21^, ^22^, ^23^, ^24^, ^25^ ^]^ This was elegantly demonstrated by Guo and colleagues who engineered doxorubicin‐loaded liposomes, grafted with ligands against both the intercellular adhesion molecule 1 (ICAM1) and epidermal growth factor receptor (EGFR), to extend mice survival beyond 14 weeks in an “early‐stage” model of breast cancer pulmonary metastasis.^[^ ^26^ ^]^ This strategy was taken even further by the group of Karathanasis who tagged liposomes with ligands against four different targets, including *α*_v_ *β*_3_ integrins, P‐selectins, EGFR and fibronectin, to demonstrate a ≈7% accumulation of the injected dose within the lung metastasis.^[^ ^27^ ^]^ A slightly different approach was followed by Jyotsana and collaborators who proposed a low dose systemic administration of dual‐targeted liposomes, decorated with anti‐E‐selectin molecules and the tumor necrosis factor‐related apoptosis‐inducing ligand (TRAIL), to intercept and kill breast cancer cells spreading from the primary tumor site following surgical resection.^[^ ^28^ ^]^ Also, the group of Yang demonstrated the autonomous intratumoral assembly of small 60 nm bio‐orthogonal particles forming larger drug depots to eradicate the primary mass and prevent its distant spreading.^[^ ^29^ ^]^ Despite these and other promising works, the specificity and biochemical stability of ligands grafted on the surface of nanocarriers as well as the spatial and temporal heterogeneity of receptors expressed on cancer cells continue to be highly questioned and, possibly, the source of mixed results presented in a variety of preclinical studies involving nanoparticles for the treatment of primary tumors and metastasis.^[^ ^30^, ^31^, ^32^ ^]^

An alternative and possibly more solid strategy to efficiently reach malignant nodules would leverage the unique vascular architecture of the lungs, which are characterized by an intricate network of small capillaries (≈5 µm) traversed by a relatively sluggish flow. Under this scenario, the authors and others have shown that sufficiently large objects, such as microparticles and red blood cells, would tend to crawl or be even transiently trapped within the pulmonary microcirculation, without compromising blood perfusion and tissue homeostasis.^[^ ^33^, ^34^, ^35^ ^]^ Indeed, this would realize ideal conditions for the localized release of molecular, macromolecular, and nano‐therapeutics from larger and slowly moving blood‐borne carriers. Following this line of thought, the authors have designed spongy and biodegradable hydrogel microparticles resulting from cross‐linking PEG chains with hydrolizable 1,4‐dithiothreitol (DTT) molecules. These microscopic hydrogel particles have been loaded with 200 nm nanoparticles carrying a variety of therapeutic and imaging agents. The geometrical, mechanical, and multifunctional features of these micro‐combinatorial hydrogel particles (µCGP) have been extensively tested in vitro before validating their therapeutic potential on a “late‐stage” murine model of TNBC metastasis to the lungs.

## 2 Results

### 2.1 Fabrication of the Multifunctional Micro‐Combinatorial Hydrogel Particles

**Figure 1:**
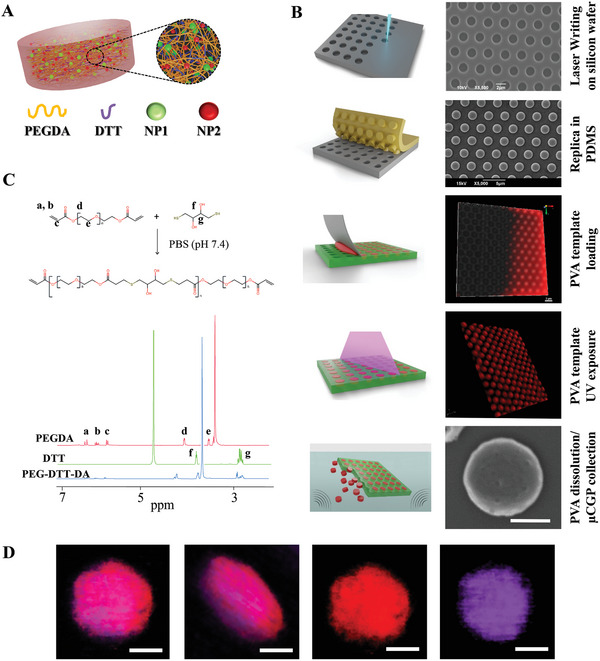
µCGP – architecture and fabrication. A) Schematic representation of a spongy µCGP comprising multiple small nanoparticles (nanoconstructs) dispersed within a porous PEG matrix; B) Sequential steps in the fabrication of µCGP presented as cartoons (left) and as SEM images of the silicon master template and PDMS template, confocal microscopy images of the PVA template loaded with the polymer paste, SEM image of an individual µCGP (scale bar: 1 µm) (right); C) The hydrolysable linker DTT reacted with PEGDA chains constituting the building block of the biodegradable µCGP matrix; NMR profile of PEGDA, DTT, and PEG‐DTT‐DA conjugates. D) Confocal images of an individual µCGP loaded with nanoconstructs carrying rhodamine‐B molecules (RhB‐SPN – purple) and directly conjugated to Ac‐PEG‐Cy5 complexes (Cy5‐PEGDA – red) (scale bar: 1 µm).

µCGP resulted from the dispersion of small nanoparticles (nanoconstructs) within the porous matrix of a microscopic PEG‐based hydrogel of precisely defined geometry. This is schematically depicted in **Figure** **1**A where multiple and different nanoconstructs (differently colored spherical beads – NP1, NP2) are shown to be dispersed within a porous polymeric matrix (orange fiber network). Lithographic microfabrication techniques were used to accurately control the geometrical attributes of µCGP, involving direct laser writing with reactive ion etching, followed by two replica molding steps, template filling and particle collection steps (Figure 1B). First, direct laser writing was employed to pattern the 2D particle cross section onto photoresist spin‐coated over a silicon wafer. This was then developed and etched into the silicon wafer via inductively coupled plasma‐reactive ion etching to a depth of ≈600 nm, resulting in an array of wells reproducing the intended geometry of µCGP. In the current configuration, these wells appear as cylinders with a ≈2000 nm diameter and a ≈600 nm depth, separated by a center‐to‐center distance of ≈4 µm (gray template in Figure 1B). This master silicon template was then replicated into a polydimethylsiloxane (PDMS) negative template, presenting a regular array of cylindrical pillars with similar dimensions (yellow template in Figure 1B). Then, the PDMS intermediate template was replicated into a water soluble poly(vinyl alcohol) (PVA) template, reproducing the original geometrical pattern of the master silicon template with a regular matrix of 2000 × 600 nm wells (green template in Figure 1B). These PVA wells were accurately filled with a polymeric paste which was rapidly cross‐linked upon exposure to UV light (red paste in Figure 1B). Finally, µCGP were collected via centrifugation upon dissolution of the PVA template in water under mild sonication. Scanning electron microscopy (SEM) and fluorescent confocal microscopy demonstrate the precise control of the µCGP size and shape in the master template as well as in its PDMS and PVA replicas (Figure 1B, right column). It is important to emphasize here that the PVA template is crucial in controlling the geometry of the resulting particles and, notably, micro hydrogels with different geometric attributes could be readily fabricated by reprogramming the laser writing step. This is documented in Figure S1 (Supporting Information), where µCGP with an elliptical, square and circular shape are presented. Indeed, the characteristic size of µCGP is dictated by the spatial resolution of the laser writing and replica‐molding processes and could span from several hundreds of nanometers to tens of microns and above. Incidentally, this soft lithographic technique is extremely flexible and has been extensively used for the fabrication of microfluidic chips and microscopic particles for a variety of applications by other groups, including the authors.^[^ ^36^, ^37^, ^38^ ^]^

In the current work, the polymeric paste consisted of PEG‐DTT‐DA, the photoinitiator 2‐Hydroxy‐4’‐(2‐hydroxyethoxy)‐2‐methylpropiophenone, and a variety of nanoconstructs. Low molecular weight PEG‐DTT‐DA chains were obtained by a Michael‐type addition reaction between 700 Da polyethylene‐glycol diacrylate (PEGDA) and DTT, in phosphate buffer saline (PBS) under gentle stirring for 3 h at 37 °C (Figure 1C). The mixing of PEGDA and DTT activates a step growth polymerization resulting in the addition of dithiol linkers between two adjacent PEGDA chains. The interposition of the hydrolysable DTT linker makes the whole µCGP biodegradable via progressive hydrolyzation. Nuclear Magnetic Resonance (NMR) spectra for PEGDA, DTT, and PEG‐DTT‐DA are shown in Figure 1C, documenting the occurrence of the reaction as described above. The showed final product decreased signals ≈6 ppm which are diagnostic for acrylate bonds upon reaction with Thiol group of DTT (signal a, b, c in the red and the blue spectra). Moreover, effective conjugation between PEGDA and DDT is confirmed by the signal at 2.8–3.4 ppm (signal f, e, and g in the green and red spectra).

To further characterize this process, the amount of free thiol groups in solution was measured over time using a Measure‐iT thiol assay, which forms a fluorescent complex upon reaction with free thiol. This fluorimetric assay confirmed that the reaction between PEGDA and DTT occurred rapidly within the first 1 h of incubation and reached a plateau of 60% already at 5 h, for the 2:1 PEGDA: DTT molar ratio used in this work (Figure S2, Supporting Information). Note that, the stoichiometric imbalance in favor of the PEGDA, ensures the presence of acrylate‐terminated chains that can therefore be photocross‐linked through their carbon double bonds and form the hydrogel. As a consequence, the resulting hydrogel network contains both photoinduced crosslinks, which are not readily degradable over months in aqueous solutions at physiologic temperature and pH, and dithiol “bridges”, which are hydrolytically labile and regulate the biodegradation of the  µCGP, as documented in the sequel.

Moreover, although in the current configuration, the authors realized micro‐hydrogels using a well‐known and clinically approved material (PEGDA), the polymeric paste could comprise a variety of water soluble synthetic and natural polymers. Similarly, the hydrolysable DTT could be readily substituted with other linkers that could respond to endogenous or exogenous signals.

The modular architecture and fabrication process of the µCGP allow for the straightforward integration of multiple functionalities by dispersing nanoconstructs carrying specific therapeutic and imaging agents, and by directly conjugating pro‐drugs to the unreacted acrylate groups of the PEG matrix. As a proof of concept, Figure 1D depicts fluorescent confocal images of an individual µCGP carrying two different fluorescent molecules, namely rhodamine‐B lipids (RhB‐DSPE), integrated in the coating of lipid‐stabilized poly(lactic‐co‐glycolic) acid (PLGA) nanoparticles (SPN); and the near‐infrared dye complexes Cy5‐PEG‐Ac directly conjugated to acrylate‐terminated polyethylene‐glycol chains. The reddish color of the µCGP (Figure 1D, left) results from the overlay between the red Cy5 signal, directly conjugated to the PEG skeleton, and the violet RhB signal, decorating the surface of nanoconstructs dispersed in the polymeric matrix (Figure 1D, right). The intense and spatially homogeneous fluorescent signal, shown in planar and tilted views for both the RhB and Cy5 dies, documents the uniform distribution of the two agents across the entire µCGP structure. Incidentally, this would confirm the accurate filling of the cylindrical wells into the PVA template during the fabrication process. To further demonstrate the modular design of the proposed particles, Figure S3 (Supporting Information) shows the µCGP carrying two different nanoconstructs, namely a RhB‐SPN (red) and a curcumin (green) loaded SPN. This flexibility makes the µCGP a combinatorial theranostic platform de facto.

Next, SPN carrying fluorophores, such as RhB (RhB‐SPN) and Cy5 (Cy5‐SPN), or the chemotherapeutic drug docetaxel (DTXL‐SPN) will be considered as the nanoconstructs for the µCGP. Note that SPNs were synthesized following procedures previously described by the authors based on a single emulsion technique and presented in the Supporting Information.^[^ ^39^ ^]^

### 2.2 Physico‐Chemical and Mechanical Features of µCGP

**Figure 2:**
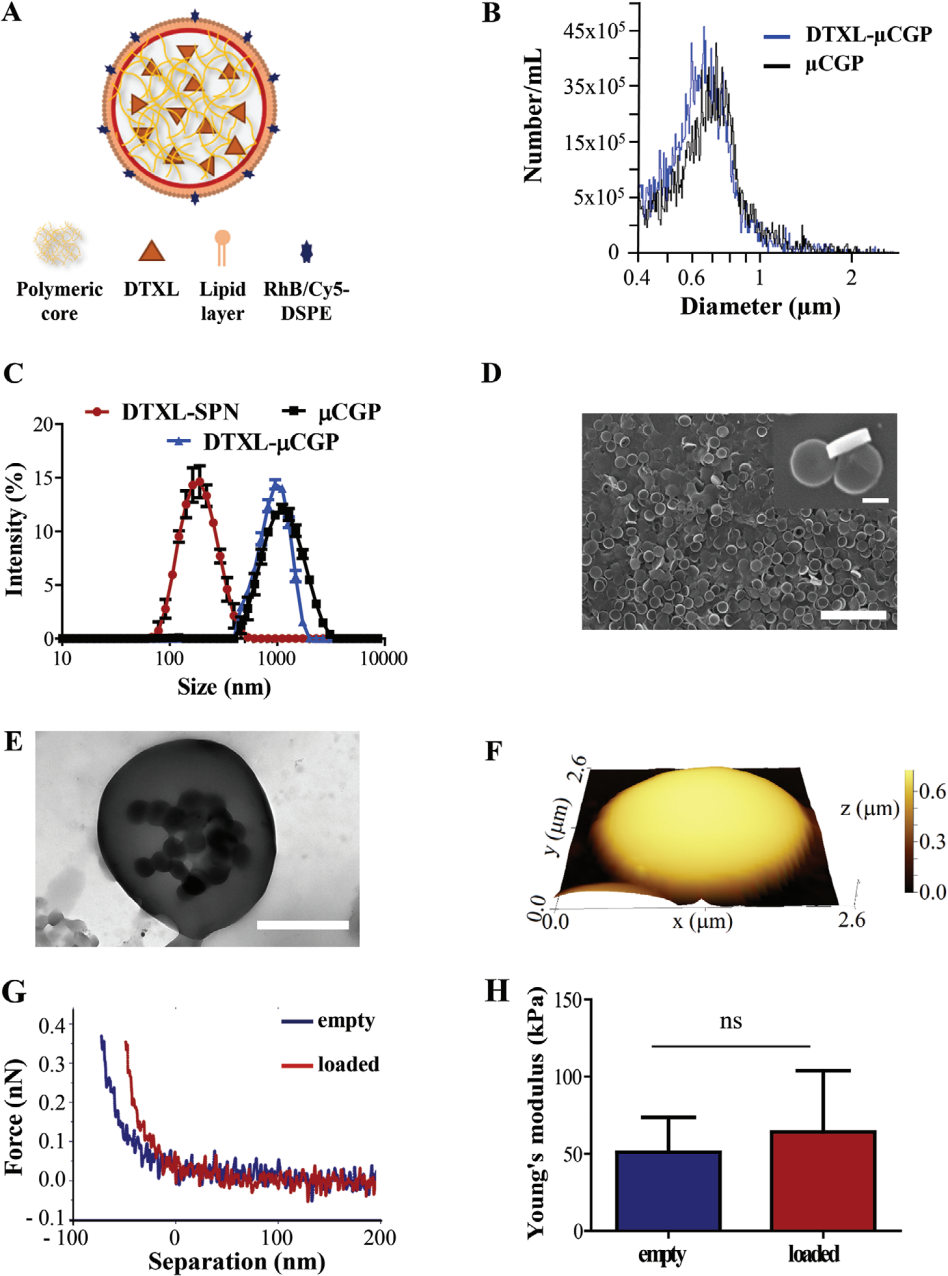
Physico‐chemical and mechanical features of µCGP. A) Schematic of the SPN dispersed within the µCGP matrix; B) Multisizer Coulter Counter measurements for empty µCGP (µCGP‐black line) and µCGP loaded with SPN carrying DTXL (DTXL‐µCGP – blue line); C) DLS measurements of SPN carrying DTXL (DTXL‐SPN – red line), empty µCGP (black line), and µCGP loaded with SPN carrying DTXL (DTXL‐µCGP – blue line). Data are expressed as mean ± s.d., *n* = 3 independent samples per particle type; D) SEM images of DTXL‐µCGP, with an inset showing front and lateral views (scale bars: 10 µm–1 µm (inset)); E) TEM image showing smaller SPN (regular, dark spots) dispersed within the polymeric matrix of µCGP (light gray) (scale bar: 1 µm); F) AFM image of a µCGP; G) Force–displacement curves for empty and loaded µCGP; H) Apparent Young's modulus for the empty and loaded of µCGP, as derived from the AFM indentation curves. Data are expressed as mean ± s.d., *n*≥10 independent samples per particle type.

A schematic representation of the fluorescently labeled and DTXL‐loaded SPN is provided in **Figure** **2**A. Next the morphological and physico‐chemical properties of µCGP were characterized via dynamic light scattering (DLS) and a multisizer Coulter Counter apparatus. The multisizer returned for the µCGP, either loaded with DTXL‐SPN or empty, spectra with peaks around 852 ± 1.54 nm and 819 ± 15 nm, respectively (Figure 2B). The DLS returned a hydrodynamic diameter of 989 ± 114 nm (polydispersity index (PDI): 0.218 ± 0.009) and 929 nm ± 306 nm (PDI: 0.178 ± 0.035) for the empty and loaded µCGP, respectively (Figure 2C, black and blue solid curves, respectively), and of 153 ± 1.7 nm (PDI: 0.153 ± 0.018) for the DTXL‐SPN (Figure 2C, red curve). The surface *ζ*‐potential was −18.7 ± 0.5 mV and −18 ± 0.6 mV for empty and loaded µCGP, respectively, and −44.6 ± 1.14 mV for the SPN. Notice that, given the actual discoidal shape of µCGP with a low aspect ratio, both the multisizer Coulter Counter and DLS do not return their exact size as these characterization techniques rely on the assumption that the objects to be measured are spherical. However, the direct comparison of the spectra allows one to conclude that the dispersion of SPN within the porous matrix of the µCGP does not alter their overall geometrical features, suggesting that SPN would be properly dispersed inside the PEG matrix rather than loosely adsorbed on the surface. This is also confirmed by the *ζ*‐potential measurements, whereby the highly negative surface charge of SPN, which is associated with the carboxylic terminations on their PEG chains, is mitigated by the PEG matrix of µCGP. Numerical values for the hydrodynamic diameters and surface *ζ*‐potential of µCGP and SPN are summarized in **Table** **1**.

**Table 1:** Table summarizing the physico‐chemical properties of SPN, empty‐µCGP, and DTXL‐µCGP in terms of hydrodynamic size, PDI, and surface *ζ*‐potential

	SPN	µCGP	DTXL‐µCGP
Multisizer [nm]	–	892 ± 1.5	819 ± 15
DLS (nm)	153 ± 1.7	989 ± 114	929 ± 306
Ζ‐pot (mV)	−44.6 ± 1.14	−18.7 ± 0.5	−18 ± 0.6
PdI	0.15 ± 0.02	0.22 ± 0.01	0.18 ± 0.03

The morphological properties of the µCGP were further investigated using three complementary microscopy techniques: SEM (Figure 2D), transmission electron microscopy (TEM) (Figure 2E) and atomic force microscopy (AFM) (Figure 2F). The SEM image confirmed the morphology of µCGP with a high electron density suggesting a tridimensional volume filled with PEG‐DTT‐DA chains. Possibly, even more interesting is the TEM image confirming the presence of small nanoconstructs within the hydrogel, corresponding to the small, regular dark spots of ≈200 nm dispersed in the light gray matrix (Figure 2E). Indeed, the SPN are more compact and realized with a high molecular weight polymer (PLGA: 36–54 kDa) as compared to the spongy structure of the 700 Da PEG µCGP. Finally, AFM allowed the authors to reconstruct the fine 3D structure of the µCGP in a Quantitative Imaging (QI) mode (Figure 2F) and to determine their mechanical properties. Representative force‐displacement curves for empty and loaded‐µCGP and the corresponding elastic moduli are shown in Figure 2G. The AFM analysis documented an apparent Young's modulus of ≈51 ± 22 and 64 ± 40 kPa for the empty and loaded µCGP, respectively (Figure 2H). Even in this case, no statically significant difference was detected, thus suggesting that the inclusion of the nanoconstructs within the PEG matrix did not affect the mechanical response of the µCGP. Indeed, Figure S4A—C (Supporting Information) support this statement by showing the effect of increasing the PEG concentration in the original polymeric paste from 5 to 50 and up to 500 mg mL^−1^. The progressive darkening of the TEM images is indicative of a higher electron density structure, confirming the higher PEG concentration. Consistently, the AFM derived apparent Young's moduli grew from 51.09 ± 22.62 to 326 ± 196, and up to 467 ± 162 kPa for the three tested PEG concentrations. It is here just important to highlight that the mechanical properties of µCGP could be readily modulated during the synthesis process by varying the concentration, molecular weight, and architecture of the PEG chains.

### 2.3 Tailoring the Biodegradation of µCGP under Different Environmental Conditions

**Figure 3:**
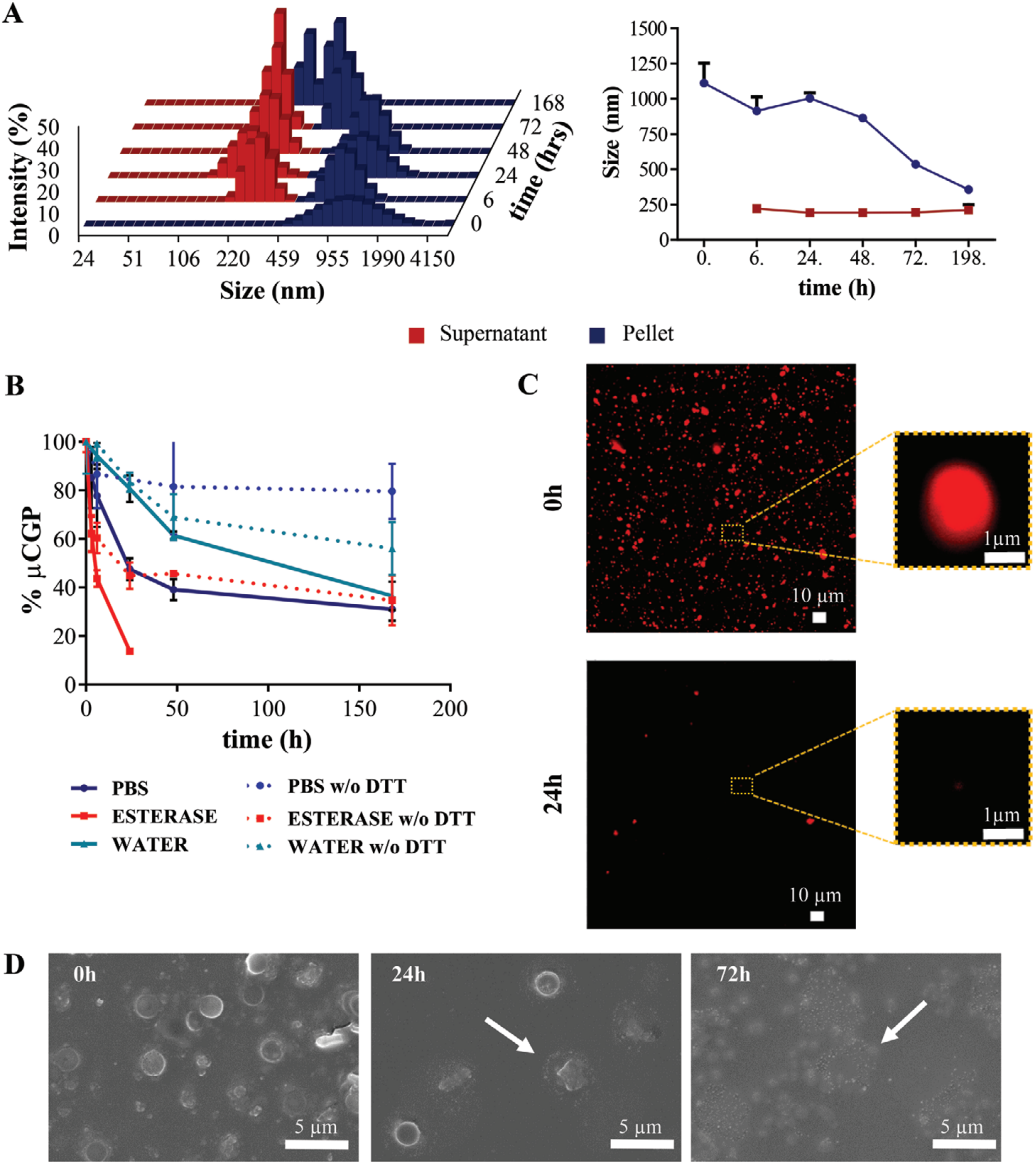
Release and degradation studies for the µCGP under different environmental conditions. A) DLS analyses showing the size distribution of particles collected in the pellet (µCGP loaded with RhB‐SPN) or supernatant (RhB‐SPN), at different time points over the course of 7 days (PBS at 37 °C) and average hydrodynamic diameters of pellet and supernatant as measured via DLS; B) Progressive degradation of hydrolysable µCGP and not hydrolysable µCGP (lacking DTT) under different conditions (PBS, DI water, PBS with esterase 10 µm), Data are expressed as mean ± s.d., *n* = 3 independent samples per each condition. C) Confocal fluorescent microscopy images of µCGP exposed to PBS with esterase (10 µm) for 24 h; D) SEM images of µCGP exposed to PBS with esterase (10 µm) for 72 h (white arrows indicate degraded µCGP).

The release of SPN from the µCGP is dictated by the passive diffusion of the ≈200 nm nanoconstructs across the slowly degrading PEG‐DTT‐DA matrix. This process was characterized using a DLS and multisizer Coulter Counter apparatus. Upon incubating µCGP in PBS at 37 °C, the supernatant and pellet of the native solution were collected via centrifugation, separating all the µCGP (pellet) from the released SPN (supernatant), and analyzed at predetermined time points. The DLS results over 7 days are shown in **Figure** **3**A. At time 0, the DLS could only detect intact µCGP, with their characteristic hydrodynamic diameter of 1112 ± 139 nm, in the pellet. As time progressed, the PEG‐DTT‐DA matrix started to degrade and release the SPN in solution. Therefore, at 6 h, two different particle populations were detected by DLS analysis: larger particles with a hydrodynamic diameter of 914.00 ± 100.64 nm were present in the pellet, corresponding to intact µCGP (Figure 3A); smaller particles with a hydrodynamic diameter of 222 ± 28 nm were observed in the supernatant, corresponding to intact SPN (Figure 3A). Notably, the two particle populations were associated to well‐defined peaks suggesting that no debris or fragments of particles could be detected in significant amounts at this early time point. Incidentally, one could also assume that SPN released within the first hours are possibly located closer to the µCGP surface and escape through its porous structure following modest or no degradation. Up to day 7, two distinct peaks were always detected, namely one in the pellet for the µCGP and one in the supernatant for the SPN, although the µCGP peak progressively moved closer to the SPN peak. However, at day 7, the µCGP peak in the pellet was almost overlapped with the SPN peak, suggesting a significant hydrolytic degradation of the native PEG‐DTT‐DA matrix. The graph close to the bar chart in Figure 3A provides the variation in hydrodynamic diameter as measured via DLS for the pellet and the supernatant at the predetermined time points. Over the first 48 h of incubation, the µCGP presented a stable hydrodynamic size with an average value ≈980 nm, consistent with the original size of these particles, which progressively decreased to 356 ± 29 nm at day 7. Also, the hydrodynamic diameter of the particle population collected in the supernatant (SPN) was equal to ≈200 nm throughout the analysis but on day 7, when the peak slightly increased because of the µCGP debris. Table S1 (Supporting Information) reports all the numerical values for both the pellet and supernatant hydrodynamic diameters up to Day 7.

To gain additional insights on the biodegradation process, the alteration of the µCGP geometry over time was further investigated via a multisizer Coulter Counter apparatus. The µCGP were resuspended in PBS, water, or in an aqueous solution containing 10 µg mL^−1^ of esterase and left at 37 °C under continuous stirring. At predetermined timepoints, aliquots of the solution were analyzed at the multisizer to quantify the number of intact µCGP (multisizer peak ≈900 nm). The resulting data are graphed in Figure 3B showing the percentage ratio between the number of intact particles at each time point and the initial number of particles. The full set of multisizer spectra are provided in Figure S5A (Supporting Information). As expected, the addition of esterase accelerated the degradation process leading to the dissolution of most µCGP (≈90%) already within the first 24 h of incubation (Figure 3B, solid red curve). This rapid enzymatic degradation must be ascribed to the presence of the acrylate ester bonds proximal to the thioether hydrolytic linkers. In PBS, the µCGP dissolution was slower and presented a biphasic behavior (Figure 3B, solid blue curve): a rapid degradation within the first 24 h, leading to a ≈50% residual µCGP, followed by a much slower degradation with a ≈30% residual µCGP at day 7. In PBS, the biodegradation of µCGP is mostly associated with the progressive hydrolysis of the dithiol linkers, which are hydrolytically labile. Finally, in water, the degradation rate was slower with a 40% of residual µCGP being still intact at day 7 post incubation (Figure 3B, solid turquoise curve). Importantly, the same experiment was conducted on µCGP lacking the DTT erodible linker, where PEGDA chains are directly crosslinked to each other (Figure 3B, dashed lines). As expected, the degradation rates were much slower with 40%, 60%, and 80% of µCGP being still intact at day 7 post incubation in esterase solution, deionized water, and PBS, respectively. The corresponding multisizer profiles are shown in Figure S5B (Supporting Information). This degradation profile was further confirmed by fluorescent and SEM images (Figure 3C,D; Figures S6 and S7, Supporting Information) showing the progressive breaking down, with reduction in size and fluorescence signal, of the µCGP over time, upon incubation in different media.

### 2.4 Cytotoxic Potential of µCGP on Triple Negative Breast Cancer Cells

**Figure 4:**
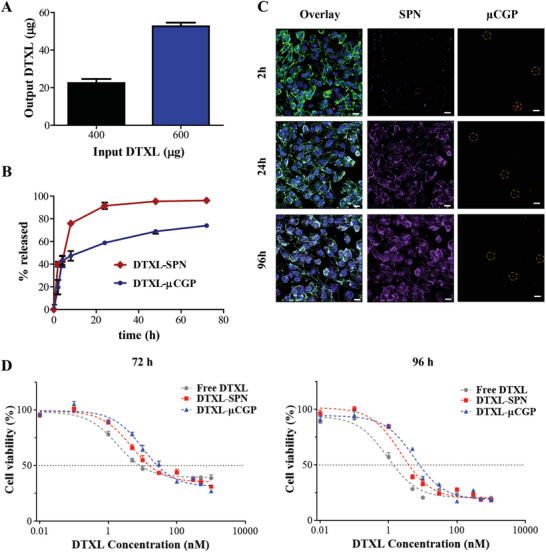
Pharmacological characterization of the µCGP on TNBC cells. A) Drug loading into µCGP for two DTXL input values (per 10 PVA templates). Data are expressed as mean ± s.d., *n* ≥ 5 independent samples per DTXL input. B) Drug release from DTXL‐SPN and DTXL‐µCGP up to 72 h (PBS, 37 °C, 4 L). Data are expressed as mean ± s.d., *n* = 3 independent samples per particle type; C) Confocal fluorescent microscopy images of Cy5‐labelled µCGP (red signal) containing RhB‐SPN (purple signal) incubated with human TNBC cells (MDA‐MB 231) for 2, 24, and 96 h. Cell nuclei stained with DAPI (blue signal) and cell cytoskeleton stained with phalloidin (green signal) (scale bar: 10 µm); D) Cell viability studies for MDA‐MB‐231 cells treated with free DTXL, DTXL‐SPN, DTXL‐µCGP for 72 and 96 hrs. Data are expressed as mean ± s.d., *n* = 10 independent samples (*R* ^2^ values ≥ 0.9).

The chemotherapeutic drug docetaxel (DTXL) was loaded into SPN, which were then dispersed into the µCGP matrix. Note that loading DTXL into SPN did not change significantly the nanoconstruct size and surface properties returning a hydrodynamic diameter of 153 ± 2 nm and a surface *ζ*‐potential of −44.6 ± 1.14 mV, as already listed in Table 1 and Figure S8A (Supporting Information). Drug loading into SPN was of ≈600 µg per batch, against an initial input of 3 mg returning an encapsulation efficiency of ≈25% (Figure S8B,C, Supporting Information). The full set of data on DTXL‐SPN is available in the Supporting Information. The dispersion of SPN (600 µg input) led to 53 ± 16 µg loaded in 10^9^ µCGP (**Figure** **4**A). Incidentally, this loading is about five times higher than previously documented by the authors using discoidal PLGA‐based nanoconstructs loaded with free DTXL or with DTXL‐conjugates (Figure S8D, Supporting Information).^[^ ^40^, ^41^ ^]^ Release studies were performed by incubating DTXL‐SPN and DTXL‐µCGP in PBS, under an infinite sink condition (4 L) and physiological environment (pH 7.4), and measuring the amounts of DTXL remaining in the particles at predetermined time points up to 72 h. Figure 4B shows the release curves as a function of time revealed by liquid chromatographic analyses. As previously reported by the authors, the ≈200 nm DTXL‐SPN released their therapeutic cargo almost entirely within the first 24 h of incubation in PBS.^[^ ^42^ ^]^ In contrast, the dispersion of DTXL‐SPN into the PEG matrix of µCGP led to a much slower release rate. Despite an initial burst release, 25% of DTXL was still detected in the DTXL‐SPN entrapped within the µCGP matrix at 72 h. At 24 h, 60% of DTXL was released from µCGP as opposed to 90% from the SPN. The released amounts of DTXL were dramatically reduced upon incubation in smaller volumes. Specifically, in a 2 mL solution, corresponding roughly to the blood volume of a small rodent, 60% of DTXL was still associated with µCGP at 72 h (Figure S8E, Supporting Information). This percentage reduces to 25% in the case of free DTXL‐SPN under the same conditions. Despite µCGP are designed to release their therapeutic cargo (SPN) from a vascular or perivascular location, which is typically characterized by normal physiological conditions, drug release has been assessed also under slightly acid conditions (pH 6.5), which typically characterize the tumor microenvironment. Results depicted in Figure S8F (Supporting Information) demonstrate, as expected, an accelerated release of DTXL from both DTXL‐SPN and DTXL‐µCGP.

The overall reduction in drug release associated with dispersing the smaller DTXL‐SPN into larger PEG hydrogel matrices could result from the steric hindrance arising at the interface between the PEG coating of the SPN and the PEG structure of µCGP. This could stabilize the SPN, diminish the influx of water and, therefore, the efflux of drug molecules. In this context, the modest initial burst release of DTXL from µCGP could be associated to the escape of peri‐superficial SPN from the PEG matrix. Indeed, there is a striking similarity between the DTXL release rates presented in Figure 4B and the biodegradation of µCGP shown in Figure 3B: within the first few hours only 20% of µCGP were loss by dissolution. Indeed, drug release and particle dissolution could be readily modulated by changing the PEG: DTT ratio as well as the nature of the erodible linker.

The direct interaction between the µCGP and human breast cancer MDA‐MB‐231 cells was assessed via confocal fluorescent microscopy. At predetermined timepoints, representative two‐dimensional and z‐stack images were acquired following particles incubation with cells at a ratio 10:1. Figures 4C and Figure S9 (Supporting Information) confirmed that the micrometric PEG µCGP (red) were not taken up by the cancer cells (cytoskeleton in green, nucleus in blue) but rather were observed to sit on or next to the cell membrane and release thereof their therapeutic cargo SPN (purple) that would slowly and continuously accumulate intracellularly. Notice that over time the red fluorescence associated with Cy5‐PEG of the µCGP reduces as the particle dissolves in PBS releasing the RhB‐labeled SPN (Figure S9, Supporting Information). The cell interaction of µCGP was also studied on murine macrophages (RAW 264.7). Results presented in Figure S10 (Supporting Information) demonstrate that µCGP (red fluorescence) are not readily uptaken by professional phagocytic cells and stay attached to the cell membrane, likely due to their shape and flexibility, as previously documented by the authors.^[^ ^43^ ^]^

Finally, it was important to assess the cytotoxic potential of µCGP on the TNBC MDA‐MB‐231 cells. This was compared to the free drug (free DTXL) and the small nanoconstructs DTXL‐SPN. The cells were exposed at different amounts of free DTXL, DTXL‐SPN and DTXL‐µCGP up to 96 h and the viability was measured via a MTT assay. The percentage of viable cells for different drug concentrations, at 24, 48, 72, and 96 h post incubation, are presented in Figure 4D and Figure S11 (Supporting Information). The response of the tumor cells to the three treatment groups followed a typical time and dose‐dependent behavior: the higher is the dose and the exposure time, the lower is the percentage of viable cells. Upon interpolating the sigmoid curves, the DTXL concentrations needed to inhibit cell growth by 50% (inhibitory concentration – IC_50_) and to induce death in 50% of the breast cancer cells (lethal dose – LD_50_) were estimated for all different conditions (Tables S2 and S3, Supporting Information). As expected, free DTXL had the lowest IC_50_ values followed by DTXL‐SPN and DTXL‐µCGP, at any given time. Specifically, at 96 h, the IC_50_ values were 0.93, 2.27, and 5.6 nm for the three treatment groups, respectively. Indeed, the lower cytotoxic potential of the DTXL‐µCGP has to be ascribed to the specific mode of action of this delivery platform: DTXL‐SPN have to first escape the µCGP matrix, then diffuse in the surrounding medium, enter the cells, and finally release free DTXL molecules upon intracellular dissolution. In agreement with this mode of action, the IC_50_ of free DTXL at 48 h (4.1 nm) was comparable to that of DTXL‐SPN at 72 h (4.75 nm) and DTXL‐µCGP at 96 h (5.6 nm). Incidentally, this data also confirms that the cytotoxic potential of DTXL is preserved across the different manipulations required for the SPN and µCGP fabrication. Finally, to demonstrate the absence of any undesired toxic effects, MTT assays were performed upon incubation of MDA‐MB‐231 cells with µCGPs loaded with empty nanoparticles (no drugs). Specifically, five particles‐to‐cell ratios were tested at 24, 48, 72, and 96 h. As depicted in Figure S12 (Supporting Information), empty µCGP resulted to be safe even at the highest tested dose (100:1 ratio) within the 96 h of incubation.

### 2.5 Therapeutic Performance in a “Late‐Stage” Pulmonary Metastasis Model

**Figure 5:**
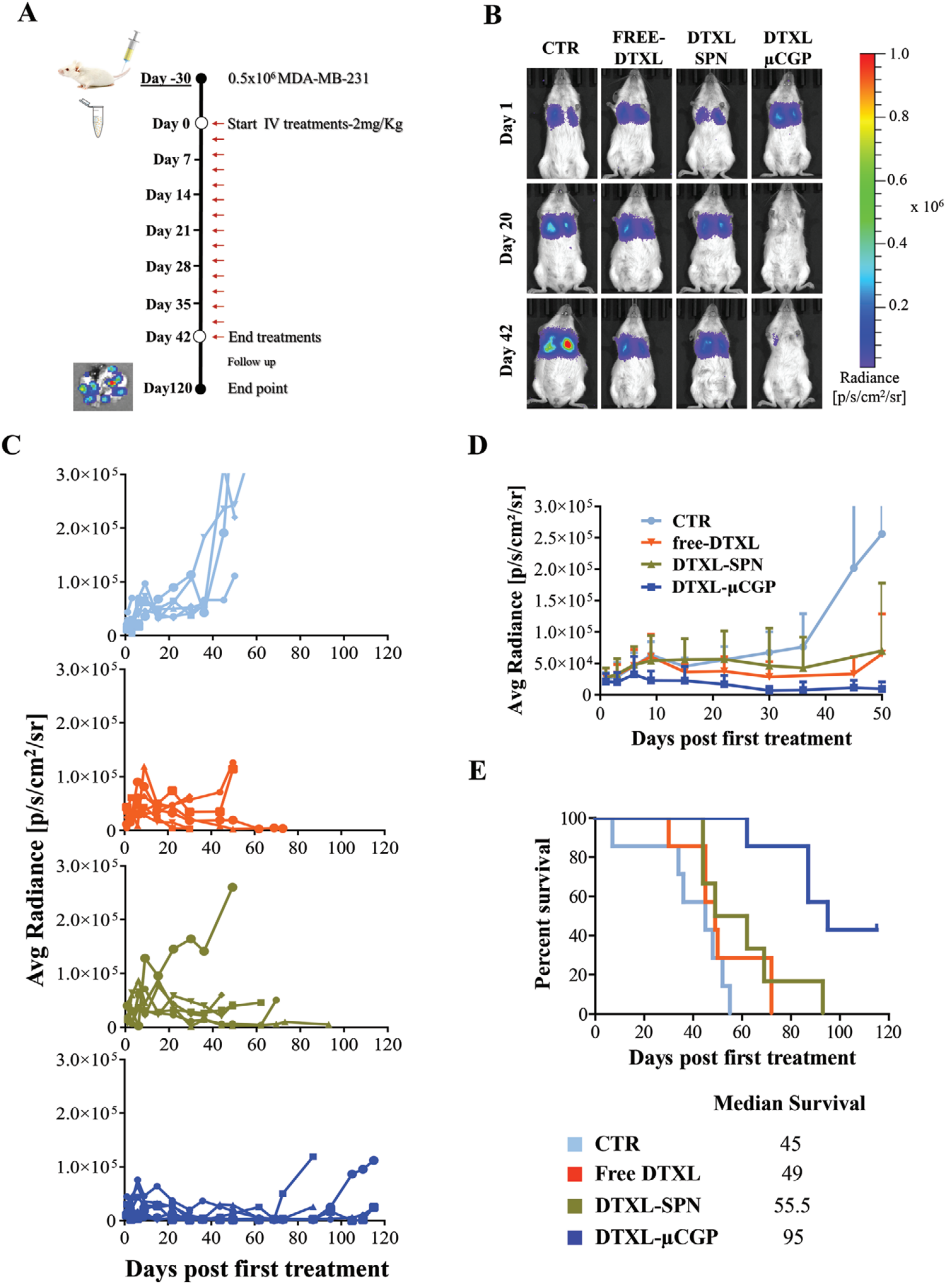
Preclinical therapeutic efficacy on TNBC late metastasis to the lungs. A) Timeline for the preclinical studies performed on NOD/SCID mice bearing lung metastasis established via the tail vein injection of 5 × 10^5^ MDA‐MB 231 cells. Red arrows indicate the timeline of the drug administrations starting at Day 0 and ending at Day 42; B) Representative bioluminescent images (BLI) at day 0 (treatment start), 20, and 42 (treatment end) for three therapeutic groups (free DTXL, DTXL‐SPN, DTXL‐µCGP – 2 mg kg^−1^ equivalent DTXL concentration) and the untreated control (saline – CTR); C) Variation of the BLI signal over time for all the mice and experimental groups; D) Direct comparison for the average BLI signal among the three therapeutic groups (free DTXL; DTXL‐SPN; DTXL‐µCGP) and control (CTR) over time. Data are expressed as mean ± s.d., *n* ≥ 6 mice per experimental group. E) Kaplan–Meier survival curves and list of median survivals.

The therapeutic performance of DTXL‐µCGP was tested in a murine model of TNBC metastasis to the lungs. This was established by injecting MDA‐MB‐231 Luc+ cells into the lateral tail vein of 6‐week‐old immune‐deficient NOD/SCID mice and letting them proliferate for 30 days until stable cancer nodules were established at different locations in the lungs. The growth of the nodules was monitored longitudinally via bioluminescence imaging (BLI), using a whole animal in‐vivo imaging system (IVIS). Once the signal associated with each nodules achieved an overall BLI surface radiance of ≈20 000 photon s^−1^ cm^−2^ sr, mice were randomly divided in four groups. **Figure** **5**A shows the timeline of the preclinical experiments. The four treatment groups included the intravenous administration, every three days for up to 42 days (15 injections), of free DTXL, DTXL‐SPN, DTXL‐µCGP at 2 mg kg^−1^ equivalent of DTXL and saline (CTR). It is here important to highlight two major differences between the present and other preclinical cancer models available in the literature: first, the proliferation of the metastasis for 30 days before treatment initiation allows one to establish a “late‐stage” metastasis model, as opposed to early metastasis models where treatment begins typically a few days after or even simultaneously with the cancer cell inoculation; second, the treatment regimen with only 2 mg kg^−1^ of DTXL and two injections per week is far less invasive in terms of dosing, injection schedule, and duration of the treatment than other approaches.^[^ ^44^, ^45^ ^]^ Figure 5B–D report on the tumor mass development over time based on the BLI signal. Specifically, Figure 5B shows representative images taken at different time points, namely day 1, 20, and 42, for mice subjected to the three treatment groups and the saline control. Notably, the BLI signal at day 1 appears to be quite similar for all the treatment groups, highlighting the reproducibility and accuracy of this “late‐stage” cancer metastasis model. The entire panel of BLI images for multiple time points and all the considered animals is included in the Figures S13–S16 (Supporting Information). Figure 5C provides growth curves for each mouse and experimental group, clearly showing the rapid and unbound proliferation of the cancer metastasis in the untreated mice (CTR).

The continuous acquisition of the BLI signal up to day 50 (8 days after treatment end) is plotted in Figure 5D for all the experimental groups and all the animals. Again, at time zero, no statistically significant difference was detected in average radiance among the four treatment groups. As expected, given the severity of the disease, the BLI signal for the CTR group (turquoise line) increased steadily throughout the entire period with an exponential growth starting ≈day 36, when the radiance was already tenfold higher than on day 1. Mice administered with free‐DTXL (orange line) and DTXL‐SPN (green line) revealed a similar trend but with slower growth rates as compared to the CTR group. Despite a moderate therapeutic benefit, both the free DTXL and DTXL‐SPN groups returned a twofold increase in radiance at day 40 as compared to day 1. This would possibly suggest that DTXL, at this low dose, tends to fuel chemoresistance and fails in treating the disease. Also, mice treated with free DTXL and DTXL‐SPN showed often clear symptoms of distress and were sacrificed before reaching day 50. Notably, post‐mortem analyses documented extensive traces of blood in the lungs of these animals, possibly associated with the rupture of blood vessels. This can be readily inferred by observing the whole lung images of Figure S17 (Supporting Information), showing multiple metastatic nodules and extensive darkening of the tissue associated with hemorrhagic processes. This is once again documenting the complexity of the disease and the severity of the “late‐stage” metastasis model here considered. Incidentally, the rupture of blood vessels could also have diminished the intratumoral accumulation of luciferin and the associated BLI signal for the free DTXL and DTXL‐SPN groups. Differently, the DTXL‐µCGP group (Figure 5D, blue line) showed a positive response to the treatment with a progressive reduction in average radiance over time that halved at day 40 as compared to day 1. The difference in average radiance among the four treatment groups was statistically significant at day 40 (*p* = 0.0235).

Perhaps, even more relevant than bioluminescence imaging is the overall mouse survival which is shown in Figure 5E. The survival curves in this Kaplan‐Meier plot document that all the saline and free‐DTXL treated mice succumbed within 55 and 70 days, respectively, from the beginning of the treatment (i.e., 30 days post tail vein inoculation of the cancer cells), returning a median overall survival of 46.5 days (less than week from treatment end). This confirms that the potent free drug DTXL at 2 mg kg^−1^, injected twice a week, cannot improve the prognosis in a model of late lung metastasis (*p* > 0.2). However, if the same drug was loaded into the SPN, mice succumbed only after 90 days, and the overall survival increased up to 55.5 days (2 weeks from treatment end). Although, the difference between free DTXL and DTXL‐SPN is not statically significant (*p* > 0.7), it shows an important trend confirming that the reformulation into nanoparticles of potent anti‐cancer drugs tends to improve their therapeutic efficacy. Finally, only mice treated with DTXL‐µCGP were observed to survive beyond 4 months, with 40% of the mice being still alive on day 120 post treatment initiation. This resulted in an overall survival of 95 days (6 weeks from treatment end), which was about twofold larger than the free DTXL (*p* = 0.0011) and DTXL‐SPN groups (*p* = 0.0078). Notably, at 90 days post treatment initiation, 50% of the DTXL‐µCGP mice were still alive whereas all the mice of the control groups had to be sacrificed or died. **Table** **2** reports the complete statistical analysis for the Kaplan–Meier survival curves performed with two different statistical tests.

**Table 2:** Table summarizing Kaplan–Meier survival curves relevant statistical analyses (*p* values)

Experimental groups	Long Rank	Wilcoxon
CTR vs free DTXL	0.2535	0.3379
CTR vs DTXL‐SPN	0.0664	0.1158
CTR vs DTXL‐µCGP	0.0001	0.0005
free DTXL vs DTXL‐µCGP	0.0011	0.0019
DTXL‐SPN vs DTXL‐µCGP	0.0078	0.0110
free DTXL vs DTXL‐SPN	0.7394	0.8314

The µCGP treatment was also well tolerated by the mice. No respiratory distress or animal loss was documented following repetitive intravenous administrations of µCGP. This was observed for at least 100 times, corresponding to at least 7 mice receiving each 15 iv injections of µCGP over the course of 30 days. Considering that pulmonary embolism is an acute event, if the systemic administration of µCGP would have triggered such a complication, clear signs of suffering and eventually death should have been observed. The absence of any complications and distress should be ascribed to the mechanical deformability of µCGP. These hydrogel particles tend to behave similarly to red blood cells, squeezing and deforming through narrow capillary beds without occluding them. Furthermore, µCGP biodegrade over time and would not accumulate for long periods of time within the lungs. Indeed, the selected PEG‐DTT molar ratio leads to a 50% biodegradation of µCGP already after 24 hrs (Figure 3). Moreover, during the preclinical therapeutic experiments, the weight of each mouse was monitored over time and no loss of weight was documented for the animals treated with µCGP for the entire duration of the experiment (Figure S18, Supporting Information). Indeed, in the first week of treatment, there was a modest decrease in weight for all experimental groups, which should be mostly attributed to the inherent toxicity of DTXL, but all the DTXL‐µCGP treated mice recovered completely their weight and continued to growth until the end of the study (day 120). Conversely, for the other groups, the animal weight was not constant and weight loss represented one of the key factors determining the sacrifice of the mice. The abnormal gain in weight observed for some mice in the saline and DTXL‐SPN groups should be associated with the formation of edema in the abdominal cavity resulting in evident enlargement of the belly.

### 2.6 Particle Biodistribution and Tissue‐Specific Drug Accumulation in a “Late‐Stage” Pulmonary Metastasis Model

**Figure 6:**
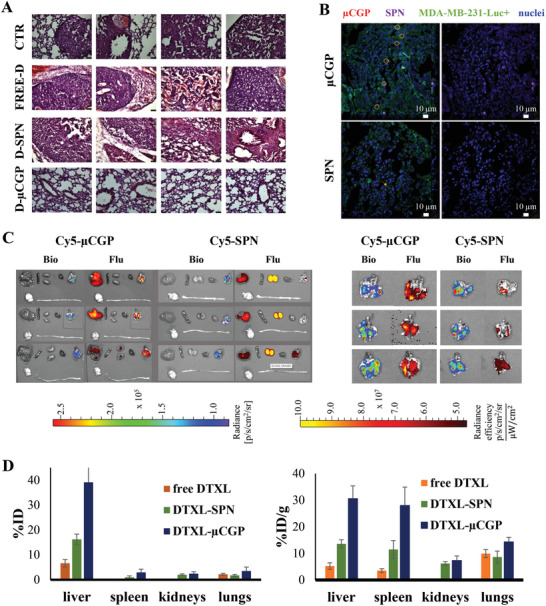
Organ specific distribution of drug and particles. A) Histological analysis by H&E staining of lungs for all the experimental groups. (Scale bar = 50 µm); B) Confocal fluorescent images of lung nodules at 24 h post injection of either Cy5‐µCGP loaded with RhB‐SPN or freely injected RhB‐SPN. (red: Cy5‐µCGP; violet: RhB‐SPN; green: MDA‐MB‐231 cytoplasm; blue: MDA‐MB‐231 nucleus); C) ex vivo bioluminescence (left) and fluorescence (right) images of the main organ (left) and lung nodules (right) at 24 h post injection of µCGP loaded with Cy5‐SPN (*n* = 3 mice per experimental group). BLI signal is associated with MDA‐MB‐231 Luc^+^ breast cancer cells; fluorescence signal is associated with Cy5‐µCGP. D) DTXL biodistribution in the major organs expressed as absolute percentage of the injected dose (%ID) and its value normalized by the mass of the tissue (%ID/g) at 24 h post systemic administration of free DTXL, DTXL‐SPN, and DTXL‐µCGP, respectively. Data are presented as the average ± SD, *n* ≥4 mice per experimental group. *p* < 0.01 for DTXL‐µCGPs versus free DTXL and DTXL‐SPN in the lungs.

Histological analyses of the lung sections, reported in **Figure** **6**A, were in general agreement with the BLI imaging and survival data. Indeed, the H&E staining identified multiple and large nodules (≥ 100 µm) in the control groups (CTR, free DTXL, and DTXL‐SPN) with respect to the mice treated with DTXL‐µCGP. Also, the characteristic alveolar structure of the lung tissue was preserved only in the DTXL‐µCGP group, while in all the other cases the uncontrolled growth of the metastatic nodules irreversibly altered the native tissue architecture. Significant amounts of red blood cells were also observed in the control experimental groups, which is consistent with the progressive damaging of the local tissue induced by the malignant progression. This is also confirmed by the whole lung pictures of Figure S17 (Supporting Information). The accumulation of systemically administered µCGP in the lungs and the metastatic nodules was assessed via four complementary techniques, including confocal microscopy images of tissue histological slices; ex‐vivo bioluminescence and fluorescence organ imaging via the IVIS system; intratissue drug accumulation via ultra‐high performance liquid chromatography – tandem mass spectrometry (UPLC‐MS/MS) analyses; and in vivo positron emission tomographic (PET) imaging.

For the first analysis, the polymeric matrix of µCGP was directly tagged with PEG‐Cy5 and loaded with RhB‐SPN and injected systemically. At 24 h post‐injection, mice were sacrificed, and the metastatic nodules were processes for histological analysis, with the cell nuclei and malignant cells stained in blue and green, respectively. Figure 6B (top row) shows µCGP (red dots) resting on the surface of the malignant cells and SPN (purple) distributed at different sites around the µCGP. Similar images were also generated for the systemically injected Cy5‐RhB (Figure 6B, bottom row). Note that, given their characteristic geometry (2000 × 600 nm discs), the µCGP were not expected to be internalized by the cells but rather sit over the cells at vascular surface acting as a local microscopic depot releasing progressively the SPN cargo. On the other hand, the systemically administered Cy5‐SPN were seen to accumulate within the malignant mass only minimally in agreement with the observed therapeutic response, highlighting that the EPR effect might not be sufficient to ensure nanomedicine accumulation in disseminated, small metastatic nodules. Additional confocal microscopy images showing particle accumulation within the sectioned metastasis are provided in Figure S19 (Supporting Information).

Using again systemically injected Cy5‐SPN and µCGP loaded with Cy5‐SPN, mice were injected, sacrificed at 24 h and their major organs, including the lungs, were ex vivo imaged via an IVIS system (Figure 6C). Aside from the liver accumulation, which is inevitable for all systemically injected particles, the Cy5‐SPN and µCGP presented very different organ deposition patterns. Cy5‐SPN returned a high fluorescent signal in the kidneys, possibly also resulting from their progressive dissolution, and a modest accumulation in the lungs. Conversely, the µCGP returned a high fluorescent signal in the lungs and a negligible accumulation in the kidneys. This would imply that the dispersion of the Cy5‐SPN within the polymeric matrix of µCGP could dramatically change the organ‐specific accumulation as compared to their direct intravenous administration. Focusing more on the lungs (Figure 6C, right), a striking spatial overlap was observed between the bioluminescence signal (malignant cells) and the fluorescent signal (particles), for both the µCGP and SPN. Importantly, the fluorescent signal associated with the µCGP was stronger than for the SPN. This would imply a convenient co‐localization of the µCGP and its therapeutic cargo with the malignant tissue.

To further support the data on the specific tissue accumulation and therapeutic response, the amount of DTXL was assessed via UPLC‐MS/MS in specific organs harvested by mice at 24 h post injection (p.i.) of free DTXL, DTXL‐SPN, and DTXL‐µCGP. The results are presented as the absolute percentage of the injected dose (%ID – Figure 6D, left) and its value normalized by the mass of the tissue (%ID/g – Figure 6D, right). At 24 h post a single intravenous injection, the amount of DTXL deposited within the lungs by the DTXL‐µCGP was about two times higher than that measured for the free‐DTXL and DTXL‐SPN (20% vs 10% ID/g) (*p* = 0.0134, Figure S20, Supporting Information). Notably, the 20% ID/g lungs was comparable with the 30% ID/g detected in the liver and spleen (2/3 lung/liver ratio). Perhaps even more instructive is the fact that the total amount of DTXL recovered in the mice injected with DTXL‐µCGP was about 50% ID against barely a 10% ID measured for the free DTXL and DTXL‐SPN. It is indeed known that the half‐life of DTXL in most organs is lower than 5 h being higher in the kidneys, followed by liver, plasma, and intestine.^[^ ^46^ ^]^ These results would therefore highlight the advantage of DTXL‐µCGP in slowly releasing and preserving the injected dose of DTXL over time. Differently, DTXL was rapidly cleared, mostly through the hepatobiliary circulation, following the systemic administration of free DTXL and DTXL‐SPN. A thorough statistical analysis on the DTXL intra‐tissue accumulation data is shown in Figure S20 (Supporting Information) demonstrating the higher DTXL amounts found in the lungs, liver, and spleen of DTXL‐µCGP over free DTXL and DTXL‐SPN treated mice (*p* = 0.0134 for the lungs, *p* < 0.0005 for liver, spleen, and kidneys).

Finally, the polymeric matrix of µCGP was radiolabeled with ^64^Cu by including Ac‐PEG‐DOTA conjugates. These radioactive particles were administered to mice bearing the breast cancer lung metastasis and imaged at 1 and 24 h p.i. PET images (Figure S21, Supporting Information) clearly showed the significant lung accumulation of the ^64^Cu‐µCGP into the lungs, most prominently at 1 h p.i. Additionally, given the inevitable accumulation of the particles in the liver, the progressive biodegradation of the µCGP and consequent loss of radioactive fragments, and the high affinity of ^64^Cu for hepatocytes, the abdominal cavity as well as the bladder returns a strong signal at both investigated time points.

## 3 Discussion

Modulating the size, shape, and mechanical stiffness of particles independently during the fabrication process is crucial for targeting different tissues and vascular districts. Several groups, including the authors, have shown that systemically administered nanometric and submicrometric spherical beads would preferentially lodge in the liver as opposed to discoidal particles that would deposit more efficiently in the lung microvasculature.^[^ ^35^, ^47^, ^48^ ^]^ Inspired by these and other observations, the groups of Muzykantov and Mitragotri demonstrated the preferential delivery of nanoparticles to the pulmonary microvasculature using ≈7.0 µm red blood cells (RBC) as vascular carriers.^[^ ^34^, ^49^, ^50^ ^]^ In the present work, the micro‐combinatorial hydrogel particles (µCGP), realized by cross‐linking PEGDA chains with DTT molecules, tend to behave similarly to red blood cell vascular transporters. Indeed, the presented results show that µCGP accumulate within the pulmonary vasculature, exploiting the fine combination of size, shape, and mechanical stiffness; and release locally small nanoparticles that would travel deeper into the diseased lung tissue. However, in contrast with the natural RBC carriers, the µCGP geometry can be precisely defined, being limited only by the spatial resolution of the laser writing process. As such, the µCGP can have a characteristic size ranging from several hundreds of nanometers to a few microns and can take up virtually any shape. Also, the deformability of the µCGP can be precisely modulated, independently from their geometry, by tuning the initial input, type, and molecular weight of the constituting polymer as well as the type and concentration of the cross‐linking agent. In the current work, by changing only the initial PEGDA input from 5 to 500 mg mL^−1^, µCGP with an apparent Young's modulus ranging from a ≈50 to 500 kPa were documented. Indeed, the proposed fabrication approach can be readily implemented for other water‐soluble polymers, such as hyaluronic acid, chitosan, or co‐polymers of PEG, and erodible linkers, that might be sensitive to endogenous stimuli – pH, cytokine, and chemokine concentrations; or exogenous energy sources – heat, light, and ultrasound.^[^ ^51^ ^]^ Therefore, the µCGP could be engineered in a variety of configurations to possibly target different vascular districts, with enhanced specificity, and address several diseases other than cancer.

Moreover, the porous matrix of the µCGP can be filled with a broad spectrum of payloads, including nanoparticles, antibodies, macromolecules and can also be directly conjugated to pro‐drugs to realize complex, patient‐specific therapeutic regimens. This feature appears to be crucial in addressing multifaceted diseases like cancer, and even more in breast cancer where a variety of therapeutic agents are currently available to target different disease subtypes. In the present work, a variety of nanoparticles were synthesized and dispersed within the µCGP matrix, including nanoparticles loaded with therapeutic molecules – docetaxel and curcumin; and imaging agents – lipid‐RhB and lipid‐Cy5; thus demonstrating the intrinsic combinatorial nature of these delivery platform. Interestingly, changing payloads and relative doses would simply ask for a modification in the original polymeric mixture before being spread over the sacrificial PVA template, without forcing to any extensive reformulation. This is indeed in striking contrast with conventional drug delivery platforms where the addition of a new agent often comes at the cost of extensive re‐engineering. Moreover, the spongy matrix of the µCGP protects the payloads from excessive losses in the circulation and provides an additional degree of freedom to dial drug release.

There is a vast literature on the successful treatment of triple negative breast cancer lung metastasis using nanomedicines and drug delivery systems. However, a direct comparison should carefully consider differences in the preclinical validation experiments too, which could reproduce “early‐stage” versus “late‐stage” metastatic diseases or even just focus on “preventive' treatments. For instance, Cao et al. developed emtansine‐loaded liposomes coated with the membrane of RAW 264.7 macrophages to demonstrate improved targeting and a significant drop in the number of metastatic nodules. This was achieved in a lung metastasis model where treatment started right after tail vein inoculation of 4T1 breast cancer cells.^[^ ^52^ ^]^ A similar "preventive” model was used by Guo et al. who targeted doxorubicin‐loaded liposomes against ICAM1 and EGFR to inhibit lung metastasis formation.^[^ ^26^ ^]^ Similarly, Mitragotri and colleagues used this approach to test the efficacy of doxorubicin‐loaded PLGA nanoparticles conjugated to the surface of erythrocytes.^[^ ^53^ ^]^ While a twofold increase in mice survival was documented for treatments starting on day 1 post cancer cell tail vein inoculation (“preventive” to “early‐stage" model), no statistically significant improvements were observed when treatment started on day 7 post inoculation (“late‐stage”). This unequivocally demonstrates that the therapeutic performance of a drug delivery system is model dependent and mature metastatic foci are more difficult to eradicate than early malignant nodules. An alternative approach is based on spontaneous lung metastasis models, where cancer cells are orthotopically inoculated in the mammary fat pad of mice and then left to grow for some time before proceeding with a resection of the primary mass. In this case, the burden of the lung metastasis is expected to correlate with the development of the primary mass before resection. This is the approach followed by Xu et al. who developed 2 µm mesoporous‐silicon discoidal particles loaded with a doxorubicin prodrug to demonstrate a ≈twofold increase in mice survival.^[^ ^54^ ^]^ More recently, the groups of Mitragotri and Muzykantov used a similar spontaneous metastasis model to validate preclinically the anti‐cancer properties of PLGA nanoparticles loaded with the immunostimulant chemokine CXCL10 and adsorbed over the membrane of erythrocytes. An ≈threefold increase in mice survival was documented for treatments starting after resecting the primary 4T1 tumor mass on day 19 post mammary fat‐pad inoculation (”early stage"). However, for the “late stage” metastasis model, obtained by resecting the primary mass on day 32 post cell inoculation, the authors documented a reduction in the number of nodules on day 50.^[^ ^50^ ^]^ Again, this continues to suggest that differences in therapeutic response could be observed with the same drug delivery platform depending on the stage of development of the disease.

In the present work, metastases were established in the lungs upon tail vein injection of MDA‐MB‐231 cells. However, treatments started only at 30 days post inoculation, allowing for the maturation of the metastatic nodules. At this same predetermined time, the organ specific deposition of the 2 µm combinatorial hydrogel particles and related therapeutic payload was assessed using complementary techniques, including fluorescent microscopy to localize µCGP and the nanoscale SPN within histological slides of the metastatic nodules; ex‐vivo optical imaging to demonstrate the preferential lung accumulation of µCGP and their co‐localization with MDA‐MB‐231 LUC^+^ malignant cells; liquid chromatographic analyses to quantify the enhancement in drug deposition in the lungs via systemic administration of µCGP over the other control groups; and the whole animal positron emission tomography imaging to show significant lung accumulation of the radiolabeled µCGP. Both the organ accumulation data and therapeutic response profiles demonstrated that re‐packaging anti‐cancer nanomedicines within µCGP could change their native biodistribution and boost their therapeutic efficacy, leading to a twofold survival increase in a “late‐stage” model of breast cancer lung metastasis. These results together with the modular architecture and flexible fabrication strategy are expected to facilitate the application of µCGP to a broad spectrum of medical conditions.

## 4 Conclusions

Micro‐combinatorial hydrogel particles (µCGP) were realized via a soft lithographic technique enabling the accurate and independent control on their geometrical (size and shape), mechanical, and pharmaceutical (loading and release) attributes during manufacturing. µCGP were shown to naturally accumulate at multiple metastatic foci developing in the pulmonary circulation and amplify thereof the specific deposition of the therapeutic cargo. This resulted in a prolonged retention of DTXL within the malignant tissue, suppression of lesion growth, and augmented survival in late‐stage triple negative breast cancer lung metastasis murine models. Given the flexible design and modular architecture of µCGP, the proposed drug delivery platform could be readily adapted to deploy complex combination therapies to cure other medical conditions.

## 5 Experimental Section

### Chemicals

Polydimethylsiloxane (PDMS) (Sylgard 184) was purchased from Dow Coming Corp (Midland, USA). Poly(vinyl alcohol) (PVA, MW 9000–10000), poly(DL‐lactide‐coglycolide) acid (PLGA, lactide:glycolide = 50:50, MW 38000–54000), poly(ethylene glycol) diacrylate (Mn 700) (PEG‐DA), 1,4‐Dithiothreitol (DTT) and 2‐hydroxy‐40‐(2‐hydroxyethoxy)‐2‐methylpropiophenone (photoinitiator), Dulbecco's Phosphate Buffered Saline (DPBS), Sucrose (BioXtra ≥ 99.5%), Modified Gill's Type II Haematoxylin, and Eosin G were purchased from Sigma‐Aldrich (Missouri, USA). Acrylate‐PEG‐Amine (AC‐PEG‐NH2, MW 2000) was purchased from Creative PEGWorks ( Chapel Hill, USA). Cyanine 5 NHS ester was purchased from Lumiprobe (Hannover, Germany). DOTA‐NHS ester was purchased from CheMatech ( Dijon, France).  1,2‐Dipalmitoyl‐sn‐glycero‐3‐phosphoethanolamine‐N‐(lissamine Rhodamine B sulfonyl) (ammonium salt) (RhB‐DSPE) was purchased from Avanti Polar Lipids (Alabama, USA). Docetaxel (DTXL) and Paclitaxel (PTXL) were purchased from Alfa Easer (Massachusetts, USA). D‐Luciferin was purchased from GoldBio (D‐Luciferin, Potassium Salt, GoldBio, Missouri, USA). 4′,6‐diamidino‐2‐phenylindole (DAPI), Red Firefly Luciferase Polyclonal Antibody, (eBioscience) and Goat Anti‐Rabbit IgG Cross‐Adsorbed Alexa 594 (Invitrogen) were all purchased from ThemoFisher Scientifics (Massachusetts, USA). Leica Frozen Section Compound FSC 22 Clear was purchased from Leica Microsystems srl, Italy.

### Fabrication of µCGP

µCGP were fabricated using soft lithographic techniques.^[^ ^55^ ^]^ First, direct laser writing was used to pattern a regular array of circles (or other predefined geometry) onto a photoresist‐coated silicon wafer. Following, the pattern was developed and then etched into the surface of the silicon wafer using inductively coupled plasma‐reactive ion etching using a Bosch‐like procedure. This resulted in a regular array of wells with a diameter *d* and a depth *h* on the silicon wafer (silicon template). If properly stored, the silicon master template can be used for years. Then, poly(dimethyl siloxane) (PDMS) was deposited and spread over the silicon template to carefully fill all the wells and cured in oven at 60 °C for 4 h. A conventional ratio between the curing agent and the elastomer of 1:10 was used. After polymerization, the resulting PDMS slab (intermediate template) was peeled off the silicon template and showed the opposite geometrical pattern of the silicon template, with a regular array of circular pillars rather than wells. Typically, the PDMS template was used for ≈6 months before generating a new one starting from the master silicon template. The third step required the careful dispersion of a 5% w/v poly(vinyl alcohol) (PVA) solution on the PDMS intermediate template and a cycle of drying in oven at 60 °C for 3 h. After drying, the PVA was peeled off the PDMS template and presented a geometrical pattern identical to that of the original template. The PVA represents the water‐soluble, sacrificial template that allows the microparticle collection upon dissolution. Starting from the PDMS template, multiple sacrificial layers could be replicated every day to allow a large particles production. 700 Da PEGDA (5 mg mL^−1^) and 1,4‐Dithiothreitol (DTT) were mixed in PBS in a 2:1 molar ratio and kept at 37 °C for 3 h to favor a Michael‐type addition reaction between PEGDA and DTT. Then, the photoinitiator 2‐Hydroxy‐4’‐ (2‐hydroxyethoxy)‐2‐methylpropiophenone) was added to the PEGDA‐DTT solution (10% v/v) that was carefully spread over the PVA template to fill the circular wells. UV light (366 nm) was used at 4 mW cm^−2^ to crosslink the PEG‐DTT‐DA chains and generate micro hydrogel particles. Finally, the PVA template was dissolved in DI water to release the µCGP, which are collected upon centrifugation.

### PEGDA‐DTT Reaction

To identify the optimal conditions for the Michael‐type addition reaction between PEGDA and DTT, the two components were added to a buffer solution (pH 7.4) and gently stirred at 37 °C for 1, 2, 3, and 24 h. At each time point, the amount of free thiol remaining in solution was quantified using a Measure‐iT thiol assay (Merck), which forms a fluorescent complex upon reaction with free thiol. Therefore, in order to assess the efficiency of the PEGDA – DTT reaction, the fluorescence intensity of the resulting samples was measured on a plate reader using a 480/20 nm excitation and 520/15 nm emission filter set. Through a standard calibration curve, the fluorescent intensity was related to the free, unreacted thiol in solution. The reaction was further characterized via Nuclear Magnetic Resoance (NMR Bruker) analysis for acrylate and thiolate species in solution.

### Physio‐Chemical Characterization

The hydrodynamic diameter and surface electrostatic charge (*ζ* potential) of µCGP were measured using a Zetasizer Nano (Malvern, UK). The µCGP size distribution and fabrication yielding was assessed through a Multisizer 4E Coulter Particle Counter (Beckman Coulter, USA). Yielding was determined as the percentage ratio between the number of particles collected via centrifugation and measured by Multisizer and the number of circular wells drawn originally in the corresponding PVA template. The geometry of µCGP was documented using a Jem‐1011 transmission electron microscope (TEM) (Jeol, Japan), upon coating with spattered carbon, and via scanning electron microscope (SEM) (Helios Nanolab 650), after 10 nm aurum coating. Fluorescent µCGP were fabricated by adding 500 µg of Cy5‐PEG‐Ac to the PEG‐DTT‐DA polymeric paste (labeling of the µCGP matrix) or by including RhodaminB‐labeled SPN (Spherical Polymeric Nanoconstructs) into the hydrogel solution of PEG‐DTT. Fluorescent µCGP were observed using an A1 confocal fluorescent microscope (Nikon).

### Mechanical Properties

The apparent Young's modulus of empty and SPN‐loaded µCGP, at a PEGDA concentration of 5 mg mL^−1^, was measured using a Nanowizard III‐JPK (Bruker) working in QI (quantitative imaging) mode in DI water. The particles had been made to adhere to the glass substrate pre‐coated with poly‐D‐lysine (PDL), for ≈20 min. A NPG‐D (Bruker) probe, with nominal spring constant of 0.06 N m^−1^ was used. The cantilever was calibrated on glass, resulting in typical optical lever sensitivity ≈17 nm V^−1^. The maximum force trigger was 0.3–0.4 nN, and the z‐range displacement was 400–500 nm.

The modeling assumed spherical tip with a radius of 50 nm, and Poisson ratio of the sample of 0.5.

First, the particles were identified under the optical microscope by a large scan with few pixels (i.e., 16 × 16), which allowed subsequent zooming in the middle of the particle, showing flat surface, to take the 32 × 32 pixel measurement of force–distance map (so‐called force–volume). A “fast scanning mode” was used along the horizontal direction as opposed to a “slow scanning mode” along the vertical direction.

Only for imaging purposes, larger size scans of up to 128 × 128 pixels (force‐distance curves) were taken, including the whole particle. The measurements were repeated in at least 7 different particles of the same type. Statistics represent the populations of the means for each measurement. The large size assumed for the tip was due to a nominal best size of 30 nm, allowing for deviations up to reaching 90 nm, according to manufacturer specifications. A similar procedure was used to assess the Young’ modulus of empty µCGP at different PEGDA concentrations, namely 5, 50, and 500 mg mL^−1^.

### Release of Nanoparticles from µCGP

To study the progressive release of SPN from the hydrogel matrix of µCGP (5 mg mL^−1^ of PEGDA), particles were resuspended in 1.5 mL of PBS 1X and left at 37 °C under stirring. At predetermined time points, namely 6, 24, 48, 72, and 168 h, the solution was centrifuged at 3250 rcf for 20 min. The supernatant was removed and replaced with fresh PBS. At this centrifugation speed, the larger µCGP were pelleted down while the released smaller SPN stayed in the supernatant.

### Degradation Studies

To study the progressive degradation of the micro hydrogel particles (µCGP), a known amount of µCGP was suspended in 1.5 mL of PBS, water, or esterase solution (10 µg mL^−1^) and left at 37 °C under stirring. At predetermined time points, namely 0, 3, 6, 24, 48, 72, and 168 h, 10 µL of the solution was further diluted in 20 mL of isotone solution and the size distribution and number of µCGP per mL was determined using a Multisizer Coulter Counter.

### Loading and Release Studies

A “direct loading” method was used to uniformly disperse SPN within the µCGP matrix. Specifically, 1 µL of SPN/polymer solution was uniformly spread over a 30 × 30 mm PVA surface containing ≈10^8^ wells. A fixed amount of DTXL loaded into SPNs (600  µg) was used to fill a number of wells returning 10^9^ µCGP. The amount of drug loaded within µCGP was calculated using a high‐performance liquid chromatography (HPLC – Agilent 1260 Infinity, Germany) and by reading the characteristic DTXL UV absorbance at 230 nm. The encapsulation efficiency was calculated considering the percentage weight ratio between the drug amount loaded within the µCGP matrix at the end of the synthesis process and the initial drug input. Release studies were performed in a volume of 4 L of buffer at controlled pH 7.4 and 37 °C to reproduce typical physiological conditions and at mild acidic conditions (pH 6.5) to reproduce the tumor microenvironment. µCGP solution (200 µL) was poured into Slide‐A‐Lyzer MINI dialysis cups with a molecular cut off of 10 kDa (Thermo Scientific) and dialyzed. At predetermined time points, µCGP were collected and dissolved in acetonitrile to read the amount of DTXL still entrapped in the matrix overtime.

### Cell Uptake Experiments

To study µCGP cell interaction/ internalization, Cy5‐PEG‐Ac was added into the polymeric matrix together with RhB‐labeled SPN. 4 × 10^4^ MDA‐MB‐231 were seeded into 8 well cover slides and incubated with the µCGP at a concentration of 10 µCGP per cell. After 2, 24, 48, 72, and 96 h, cells were fixed using 4% paraformaldehyde. Actin was stained in green using phalloidin (thermo) and nuclei using DAPI following vendors indications. µCGP uptake was dissected via 2D images and Z‐stacks acquired on a Nikon confocal fluorescent microscope.

### Cell Viability Studies

3‐(4,5‐ dimethyl thiazolyl)‐2,5‐diphenyltetrazolium bromide (MTT‐Sigma Aldrich) tests were performed on MDA‐MB 231 triple negative breast cancer cells to assess the in vitro therapeutic efficacy of µCGP as compared to chemotherapeutics administered in their free form or loaded within conventional SNP. Cells were obtained from the American Type Culture Collection (ATCC). Cells were cultured in Eagle's minimal essential medium (EMEM) (ATCC, USA) completed with 10% fetal bovine serum (FBS) (Gibco, Thermo Fisher Scientific, USA), 1% penicillin/streptomycin (Sigma‐Aldrich, USA), under a humid atmosphere (37 °C, 5% CO2, 95% air). To perform the viability tests, 10^4^ cells were seeded into 96‐well plates (cell density of 33000 cells cm^−2^) and the day after treated with EMEM medium containing growing doses of free DTXL, DTXL‐SPN, and DTXL‐μCGP (0.1–1000 nm). After 24, 48, 72, and 96 h, the treatment solution was removed and replaced by MTT solutions, according to the manufacturer's instructions. The resulting formazan crystals were then dissolved in ethanol (100 µL per well) and the absorbance was read at 570 nm using a microplate reader (Tecan, CH). Cell viability was normalized to that of untreated cells.

### Histological Analysis

After harvesting, organs were fixed in 4% paraformaldehyde (PFA) solution in PBS (Santa Cruz Biotechnology, Inc., Heidelberg, Germany) for 1 day at 4 °C, immersed in a 30% (w/v) sucrose solution in PBS for 2 days at 4 °C, and frozen by vapor of liquid nitrogen, then stored at −80 °C until sectioning. Organs were embedded into Frozen Section Compound (Leica FSC 22 Clear, Leica Microsystems srl, Italy) and 20 µm slices were prepared using cryostat instrument. The sections were then placed on HistoBond® microscope slides (Marienfeld, Germany) and stained with Haematoxylin‐Eosin (Modified Gill's Type II Haematoxylin; Eosin G) or 4’,6‐diamidion‐2‐phenylindole (DAPI) and Luciferase antibody (Primary Antibody, Red Firefly Luciferase Polyclonal Antibody, eBioscience, 1:50; Secondary Antibody, Goat Anti‐Rabbit IgG Cross‐Adsorbed Alexa 594, Invitrogen, 1:1000) for tumor recognition. Slices were imaged using confocal microscopy (Nikon A1).

### µCGP Biodistribution

µCGP loaded with Cy5‐SPN were used to assess the particle biodistribution upon intravenous injection using a whole animal optical imaging system (IVIS). Free Cy5‐SPN were used as a control group. Nude mice (CD1 Nu/Nu) bearing lung metastasis were systemically administered with fluorescent µCGP and fluorescent SPN and monitored longitudinally by acquiring images at 30 min, 1, 3, 6, and 24 h post injection (p.i.) and post‐mortem of the harvested organs. To perform quantitative biodistribution, NOD/SCID mice bearing lung metastasis received one single injection of 2 mg kg^−1^ free DTXL, DTXL‐SPN, and DTXL‐µCGP. Each formulation was diluted in saline solution and administered through retro‐orbital injection. Mice were sacrificed 24 h post injection and the collected organs were weighed and stored at −80 °C until further analysis. Liquid chromatography – tandem mass spectrometry (LC‐MS/MS) was used to quantify the amount of DTXL accumulated into the organs. Specifically, organs were washed, diluted at a 1:10 w/v ratio in PBS plus 1% of protease inhibitor and homogenized by using a T10 basic ultraturrax (IKA Technology). For all the samples, 200 µL of tissue homogenate were crashed with an equal volume of acetonitrile containing paclitaxel (300 nm), as internal standard, vortexed for 1 min and centrifuged at 3000 × g at 4 °C for 10 min. The quantification of DTXL was performed using a XEVO TQMS triple quadrupole mass spectrometer, equipped with an electrospray ion source and coupled with an Acquity UPLC System (both purchased from Waters). Spectra were acquired in positive ion mode (ESI+). For the chromatographic separation, an Acquity BEH C18 (2.1 mm × 50 mm, 1.7 µm particle size) column was used with the following eluents: A = water + 0.1% formic acid; B = acetonitrile + 0.1% formic acid. The analytes (DTXL and internal standard paclitaxel) were separated and eluted from the matrix using a linear gradient of eluent B in A, from 10% to 90% in 5 min. The flow rate was kept at 0.5 mL min^−1^. The column temperature was maintained at 45 °C and the injection volume was set to 7 µL. DTXL quantification was performed in MRM mode (multiple reaction monitoring) by selecting compound‐specific precursor to fragment transitions.

### Tumor Model and Therapeutic Experiments

All animal experiments were performed according to the guidelines established by the European Communities Council Directive (Directive 2010/63/EU of 22 September 2010) and approved by the National Council on Animal Care of the Italian Ministry of Health. All efforts were made to minimize animal suffering and use the lowest possible number of animals required to produce statistical relevant results, according to the “3Rs concept”. To develop an experimental model of lung metastasis, 6‐week‐old female NOD/SCID mice (Charles River, Calco, Italy) were used. Animals were grouped in ventilated cages and able to freely access food and water. They were maintained under controlled conditions: temperature (21 ± 2 °C), relative humidity (50 ± 10%), and light (12 h of light and 12 h of dark). For the injection, trypsinized 5 × 10^5^ MDA‐MB‐231 luciferin positive cells were resuspended in cold PBS solution. A total of 100 µL of PBS was systemically injected into the lateral tail vein and cells were left to proliferate for 30 days to establish stable lung nodules (*late‐metastasis model*). The growth of the nodules was followed by IVIS spectrum system every 2 days, upon intraperitoneal injection of D‐luciferin, potassium salt (GoldBio) at a dose of 150 mg kg^−1^. Upon reaching an average radiance of ≈20 000 photon s^−1^ cm^−2^ sr, mice were randomly divided in four experimental groups (*n* ≥ 6): the “CTR” group, including mice injected with PBS; the “free‐DTXL” group, including mice injected with a DTXL solution (2 mg kg^−1^); the “DTXL‐SPN” group, including mice treated with 2 mg kg^−1^ of DTXL‐loaded SPN and the “DTXL‐µCGP” group including mice treated with 2 mg kg^−1^ of DTXL‐SPN loaded µCGP. In all the cases, the agent administration was performed intravenously every 3 days for up to 42 days, returning a total number of injections equals to 15. Note that the DTXL administered dose of 2 mg kg^−1^ was significantly lower than that conventionally used in pre‐clinical experiments. The therapeutic efficacy of the different treatments was evaluated by whole animal optical imaging (IVIS). All mice were euthanized when they became moribund, or nodules radiance exceeded 10^5^ values. Survival was monitored and plotted using the Kaplan–Meier method.

### Statistical Analysis

All data were processed using Excel 2010 software (Microsoft) and GraphPad PRISM. Results are expressed as the mean plus standard deviation. Statistical analyses on in vivo experiments were performed using Student's *t*‐test. Log‐rank test was used to test the significance of different survival curves. The *p* values of < 0.05 (*), < 0.01 (**), and < 0.001 (***) were statistically significant.

### Ethics Declarations

All animal experiments were performed according to the guidelines established by the European Communities Council Directive (Directive 2010/63/EU of 22 September 2010) and approved by the National Council on Animal Care of the Italian Ministry of Health.

## Conflict of Interest

The authors declare no competing interests. A.L.P. and P.D. are co‐inventors on a patent application “Combinatorial hydrogel nanoconstructs for drug delivery and imaging in cancer and other disease” (IT102022000002486) filed by the Fondazione Istituto Italiano di Tecnologia. The remaining authors declare no competing interests.

## Author Contributions

A.L.P. and P.D. conceived the idea and designed all the experiments. A.L.P. realized the different type of µCGP and performed all the in vitro and in vivo experiments, analyzed all the data, performed the statistical analysis. D.D.M. realized the DTXL‐SPN for the imaging and therapeutic studies and conducted the histological analyses. M.F. and P.G. performed the nuclear imaging experiments. R.S. helped perform the optical imaging experiments and prepared the samples for histological analysis. A.F. conducted the NMR characterization and realized the PDMS templates for the µCGP synthesis. T.L.M. synthesized the 2 µm silicon master template. L.B. and A.A. performed liquid chromatography–mass spectrometry analyses. M.S. performed the atomic force microscopy characterization. A.L.P. and P.D. wrote the manuscript. P.D. supervised the whole project. All the authors read and discussed the data.

## Supporting information

Supporting Information

## Data Availability

The data that support the findings of this study are available from the corresponding author upon reasonable request.
