# Supplementary material for: Boosting the Potential of Chemotherapy in Advanced Breast Cancer Lung Metastasis via Micro‐Combinatorial Hydrogel Particles
Source: Adv Sci (Weinh). 2023 Jan 22;10(10):2205223. doi: 10.1002/advs.202205223 (PMC10074128; doi:10.1002/advs.202205223)
Supplement: Supplementary file 1 — Supporting Information [file ADVS-10-2205223-s001.pdf]

ADVANCED SCIENCE
Open Access

Supporting Information

for *Adv. Sci.*, DOI 10.1002/advs.202205223

Boosting the Potential of Chemotherapy in Advanced Breast Cancer Lung Metastasis via Micro-Combinatorial Hydrogel Particles

*Anna Lisa Palange, Daniele Di Mascolo, Miguel Ferreira, Peter J. Gawne, Raffaele Spanò, Alessia Felici, Luca Bono, Thomas Lee Moore, Marco Salerno, Andrea Armirotti and Paolo Decuzzi**

Supporting Information

Boosting the Potential of Chemotherapy in Advanced Breast Cancer Lung Metastasis via micro-Combinatorial Hydrogel Particles

Anna Lisa Palange[1], Daniele Di Mascolo[1], Miguel Ferreira[1,♠], Peter J. Gawne[1], Raffaele Spanò[1], Alessia Felici[1,♠], Luca Bono[2], Thomas Lee Moore[1], Marco Salerno[3], Andrea Armirotti[2], Paolo Decuzzi[1,♣]

[1] Laboratory of Nanotechnology for Precision Medicine

[2] Analytical Chemistry Facility

[3] Materials Characterization Facility

Fondazione Istituto Italiano di Tecnologia, Via Morego 30, 16163, Genoa, (Italy)

♣ Corresponding author: Paolo Decuzzi, PhD; paolo.decuzzi@iit.it

♦ Current addresses:

Miguel Ferreira, PhD; mferreira@mgh.harvard.edu, Harvard Medical School, Department of Radiology, Massachusetts General Hospital, Boston, MA, USA

Alessia Felici, PhD; afelici@stanford.edu Division of Oncology, Department of Medicine and Department of Pathology, Stanford University School of Medicine, Stanford, CA, USA

SUPPORTING METHODS

Fabrication of Spherical Polymeric Nanoconstructs (SPN). Spherical polymeric nanoconstructs (SPN) were synthesized by a sonication-evaporation technique already described by the authors[1]. Briefly, 10 mg of PLGA, 1,2-dipalmitoyl-sn-glycero-3-phosphocholine (DPPC), and DTXL were mixed in chloroform (oil phase). 1,2-distearoyl-sn-glycero-3-phosphoethanolamine-N-[carboxy(polyethylene glycol)-2000] (DSPE-PEG) was dispersed in a aqueous solution of 4% ethanol (water phase). The lipids DPPC and DSPE-PEG together constituted the 20% in weight of the PLGA mass used. DTXL was the 30% w/w of the polymer content. The oil phase was then added dropwise to the aqueous phase under probe sonication. The resulting emulsion was transferred in a reduced pressure environment under continuous stirring for organic solvent evaporation. SPN were then purified by sequential centrifugation steps. A first step was performed at 250 x *g* for 2 min to remove any possible debris deriving from the synthesis process. Further centrifugation steps were performed at 15,000 x *g* for 15 min to remove all the materials loosely bound to the SPN surface or free in solution. SPN labeled with Rhodamine-B (RhB-SPN), Cy5-lipid (Cy5-SPN) and DOTA lipid (DOTA-SPN) were synthesized following the same protocol but substituting 20 µg of DPPC with a 1,2-dioleoyl-sn-glycero-3-phosphoethanolamine-N-(lissamine Rhodamine-B sulfonyl) (RhB-lipid), Cy5-modified 1,2-distearoyl-sn-glycero-3-phosphoethanolamine-N-[amino(polyethylene glycol)-2000], and 1,2-dioleoyl-sn-glycero-3-phosphoethanolamine-N-DOTA, respectively.

Physico-chemical characterization of SPN. The hydrodynamic size and surface ζ-potential of SPN were quantified using a dynamic light scattering analysis (Nano ZS, Malvern, UK). Specifically, after purification, SPN were dispersed in Milli-Q water, and then a small volume of SPN solution (i.e., 20 µl) was added to 1 ml of Milli-Q water and analyzed. For the ζ-potential measurements, Folded Capillary Zeta Cells (Malvern, UK) were used.

Drug loading and release for SPN. Drug loading and encapsulation efficiency into SPN were quantified via HPLC (1260 Infinity Agilent Technology, U.S.A.) analyses (n = 10) using an acetonitrile:water (1:1) solution to disrupt the nanoparticles and fully release the entrapped

drug molecules. Loading is defined as the ratio between the mass of the loaded drug and the total mass of the nanoparticles. The encapsulation efficiency is defined as the percentage ratio between the mass of the loaded drug and the input drug.

For drug release studies, DTXL absorbance at 230 nm was considered. Specifically, SPN were dispersed in PBS and placed in dialysis cups (Slide-A-Lyzer MINI dialysis microtubes with a molecular cutoff of 10 kDa, Thermo Scientific) that were then exposed to PBS (4L), as the receptive phase. At different time points, 3 cups were removed from the solution, and their content was centrifuged to collect the SPN. These were then dissolved in an acetonitrile:water (1:1) solution before HPLC analysis. Release data are shown in the form of percentage cumulative release, which was obtained by adding the DTXL amount measured at a specific time point to all the previous time points and normalizing the resulting values for the initial amount of DTXL loaded into SPN.

NMR analysis for PEGDA-DTT conjugation. 5 mg of the PEGDA, DTT and PEGDA-DTT samples were dissolved in 0.5 ml of D2O and transferred into 5 mm SampleJet disposable tubes (Bruker). NMR experiments were performed at 298 K on a Bruker Avance III 600 MHz spectrometer equipped with 5 mm QCI cryoprobe with z shielded pulsed-field gradient coil, and operating at 600.13 MHz. 1H NMR spectra were acquired, after having applied a 30 degree of flip angle, with 16 transients, 65,536 points of digitalization, 1s of inter-pulses delay over a spectral width of 20.03 ppm (offset positioned at 6.18 ppm).

SUPPLEMENTARY RESULTS

Morphological characterization of μCGP with different geometries. Following the synthesis process described in the main text, μCGP were also realized with an elliptical and a quadrangular base as well as with templates carrying circular wells of different diameters, using the same polymeric paste. All μCGP were loaded with SPN carrying the small naturally fluorescent molecule curcumin (CURC-SPN). **Figure S1** reports confocal images of the loaded PVA template, TEM images of an individual μCGP, and the DLS spectrum of the three different geometries (circular, elliptical, quadrangular). Imaging analyses confirmed the accurate control on the particle geometry. The DLS data showed a uniform and monodispersed

population with an average size of 819 ± 15, 1,540 ± 130, 716 ± 106 for the circular, elliptical, and quadrangular µCGP, respectively.

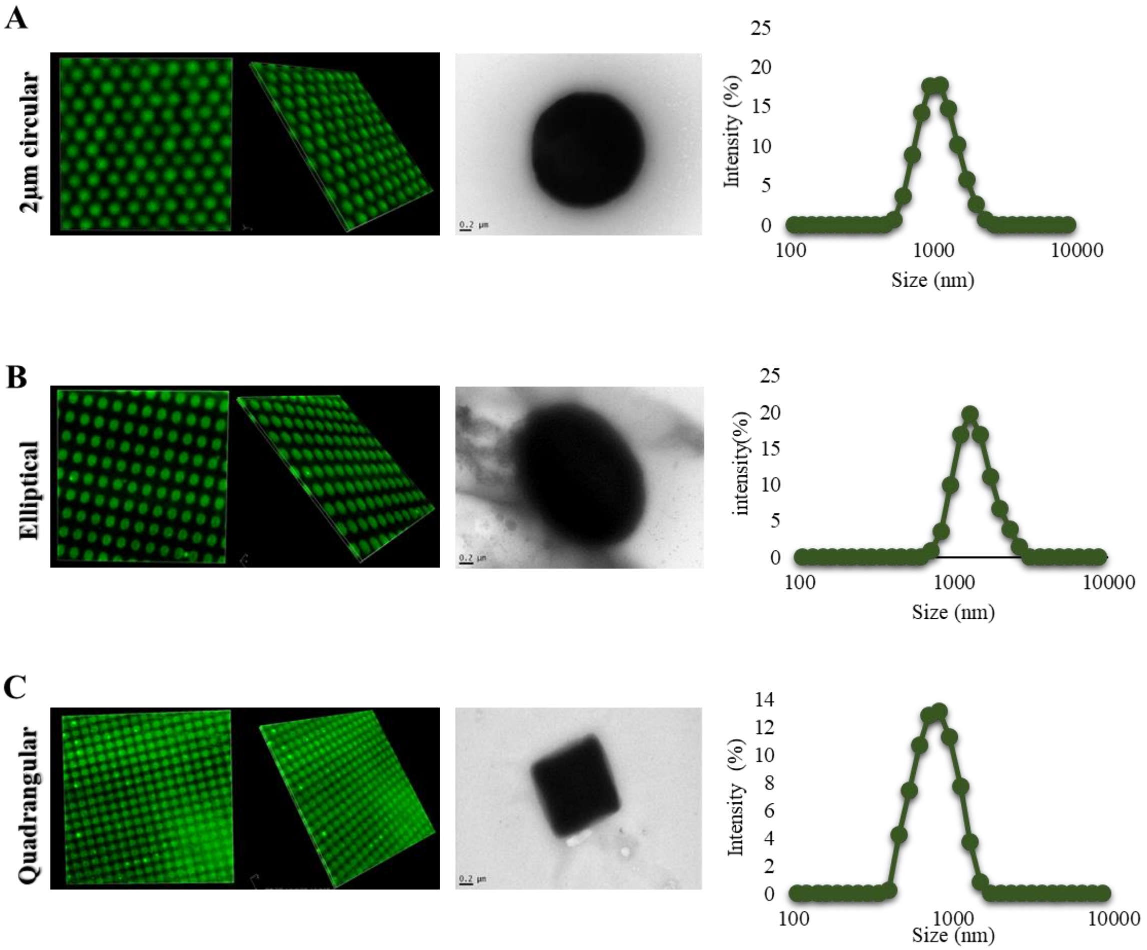

Figure S1. **µCGP with different geometries**. Confocal, TEM, and DLS analysis of µCGP with a circular, elliptical, and quadrangular base.

Characterization of the PEGDA-DTT conjugation.

The chemical structure of the PEGDA-DTT product have been examined by 1H-NMR as described in the main text. To further characterize the PEG-diacrylate (PEGDA) reaction with DTT, DTT was added to a PEGDA solution at a 1:2 molar ratio. The solution was allowed to react for 60 min at 37°C. The amount of free thiol remaining in solution after each reaction was characterized using the Measure-iT thiol assay (Invitrogen Carlsbad, CA), which forms a fluorescent complex upon reaction with free thiol. The fluorescence intensity of the samples was measured on a plate reader using a 480/20 nm excitation and 520/15 nm emission filter set, and

related to the free thiol concentration via a standard curve. As depicted in **Figure S2**, the reaction of PEGDA with DTT was rapid and efficient. When DTT was introduced into buffered solutions containing PEGDA and allowed to react for 60 min, the thiol concentration rapidly dropped to 40% already after 1h. This residual concentration was then observed to stay constant for up to 24h, suggesting that not all the DTT was reacted with the PEGDA chains. Consequently, free acrylate groups not reacted with DTT would be available in the polymeric matrix of the µCGP.

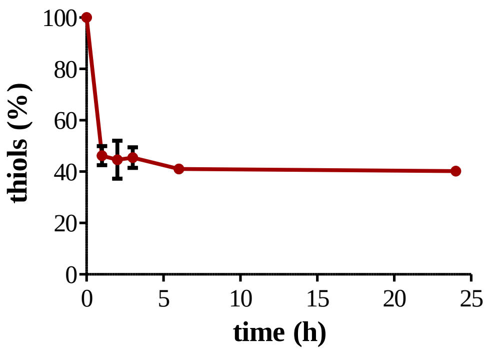

Figure S2. **PEG-DTT conjugates**. Thiols fluorimetric assay showing the reduction of free thiols group during the PEG-DTT reaction at 37 °C. Data are expressed as mean ± s.d., n=3 independent reactions.

Combining different nanoparticles in µCGP. µCGP were co-loaded with two different SPN, one tagged with the RhB fluorophore (red) and one carrying curcumin (green). The **Figures S3A, B** show confocal fluorescent images of the PVA template loaded with the polymeric paste resulting from the mixture of PEG-DTT, RhB-SPN, and CURC-SPN. The presence of green and red fluorescence signals confirmed the simultaneous dispersion of the two different SPN within the µCGP matrix. The z-stack images (**Figure S3C**) taken over different planes of the µCGP confirmed the presence of the two SPN throughout the microgel structure. Notice that following this approach different nanoparticles, antibodies, and macromolecules, carrying both imaging and therapeutic agents, can be combined within the polymer matrix of µCGP.

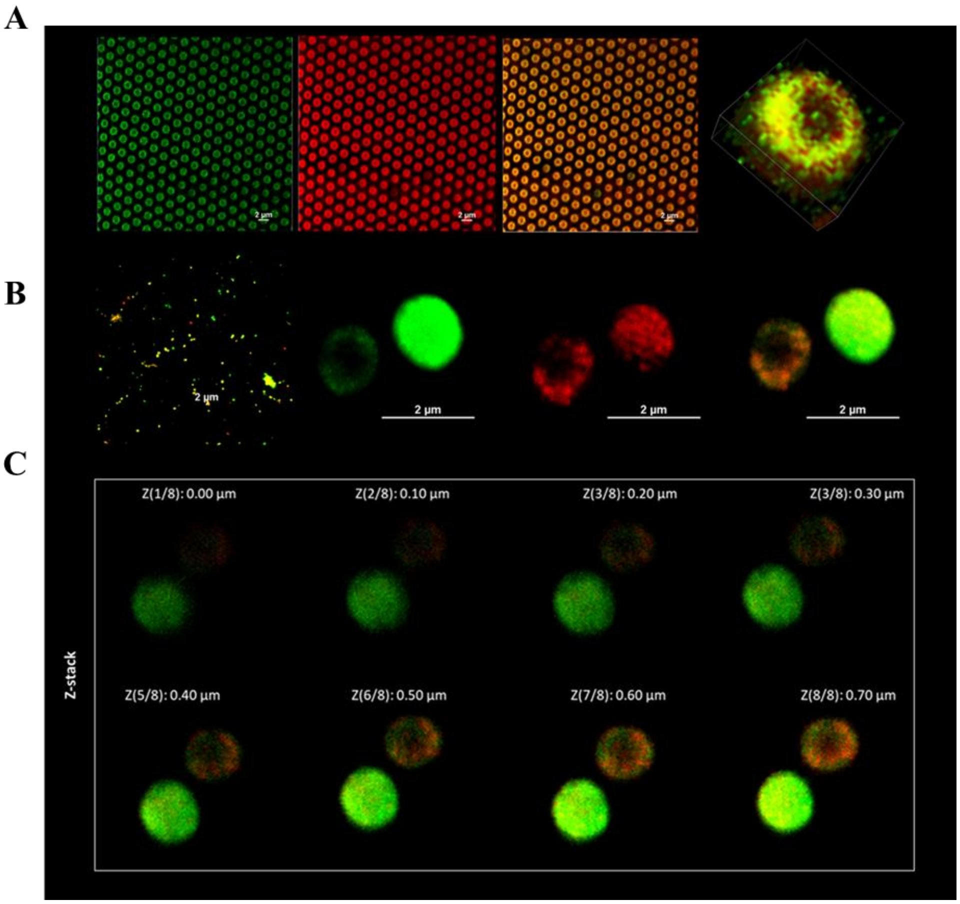

Figure S3. Combinatorial hydrogel particles. A. Confocal images of PVA templates loaded with a PEGDA polymeric mixture including RhB-SPN (red) and CURC-SPN (green). **B**. Multiple (left) and individual µCGP loaded with RhB-SPN (red) and CURC-SPN (green). **C**. z-stack analyses showing SPN distribution across the full thickness of the µCGP matrix.

µCGP mechanical characterization. Transmission electron microscopy (TEM) and atomic force microscopy (AFM) imaging analyses were performed to characterize the mechanical properties of µCGP realized with different PEG concentrations, ranging from 5 to 500 mg/mL. The larger the PEGDA concentration, the larger the stiffness of the µCGP. **Figure S4A** shows how the increased polymer concentration leads to a more electron dense particle with a coloration varying from light gray to dark gray and black in the TEM images. The actual mechanical stiffness of the µCGP was quantified via AFM analyses. **Figure S4B, C** show AFM 3D images of the microgel particles and reports the apparent Young's modulus for each PEG concentrations, reaching the value of 500 kPa for the 500 mg/mL configuration. Indeed, the

stiffness of the µCGP can be modulated during the synthesis process by varying the concentration of the polymer.

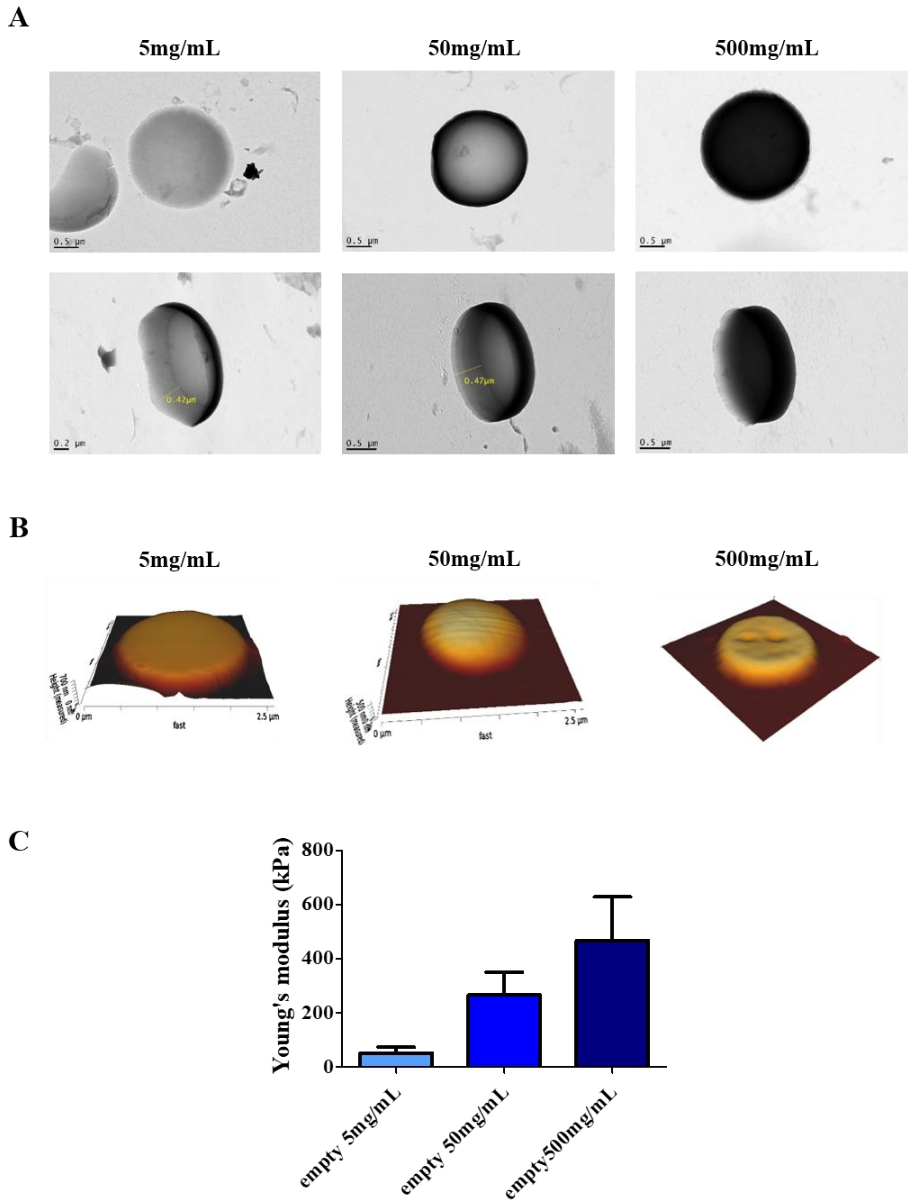

Figure S4. A. TEM images of µCGP with increasing PEGDA concentrations, namely 5, 50, and 500 mg/mL. **B-C**. AFM images and apparent Young's modulus for µCGP with increasing PEGDA concentrations. Data are expressed as mean ± s.d., n≥10 independent samples per particle type.

µCGP biodegradation. The degradation rate of the µCGP polymeric matrix was assessed by monitoring the particle geometrical features (size distribution) via a Multisizer Coulter Counter

apparatus. Specifically, µCGP were suspended in 1.5 mL of PBS 1X, DI water, or a solution containing 10 µg/mL of esterase (three different environmental conditions) and left at 37 °C under stirring. At each time point, namely 0, 3, 6, 24, 48 and 168h, 20 µL of solution was diluted into 20 mL of isotone and characterized at the Multisizer to count the number of µCGP per mL and assess their geometrical features. **Figure S5A** shows representative Multisizer profiles at 0 and 24h for all the tested conditions. Incubation in esterase determined a prompt degradation of µCGP with a 90% loss in particles already at 24h. The rapid enzymatic degradation of µCGP is related to the presence of the acrylate ester bonds proximal to the thioether hydrolytic bridges. µCGP degradation in PBS was slower with a loss of about 50% of the particles at 24h. Under this condition, µCGP degrade for the progressive hydrolysis of the dithiol 'bridges', which are hydrolytically labile. The degradation in PBS continued overtime reaching a loss in particle of 70% at 168h post incubation. In DI water, the degradation rate was even slower returning only a 40% loss in particles at 24h. The same experiments were also conducted for µCGP consisting of PEGDA only (no DTT) (**Figure S5B)**. Indeed, the particle degradation is much slower than for the PEG-DTT µCGP supporting the notion that the addition of the thiol bridges is fundamental for obtaining biodegradable microhydrogels. Degradation data were further confirmed by confocal (**Figure S6**) and SEM imaging (**Figure S7**). In particular, the confocal analysis shows how in water, the number of particles and the fluorescent intensity of µCGP was not significantly altered at 24h post incubation. On the other hand, in PBS, a 50% decrease in the number of particles was appreciated, while, in esterase, a massive drop in fluorescence and number of particles was observed after 24h, when it is evident that particles lose their native discoidal shape. Even more informative are the pictures taken at higher magnification in **Figure S6B** that show the progressive degradation of µCGP in an esterase solution: the particle morphology is completely lost at 72h.

Time (hrs)	Pellet (nm)	Supernatant (nm)
0	1112.3 ± 14	-
6	914 ± 100.64	222.33 ± 28
24	1004.8 ± 38.3	192.67 ± 14.2
48	864 ± 12.77	192 ± 21.59
72	535.67 ± 21	195 ± 5
168	356.33 ± 29.1	206 ± 50.91

Table S1. SPN release from the micro-Combinatorial hydroGel Particles. Table provides average hydrodynamic diameters of particles collected in the pellet (μCGP loaded with RhB-SPN) or surnatant (RhB-SPN), at different time points over the course of 7 days (PBS at 37°C) as measured via DLS.

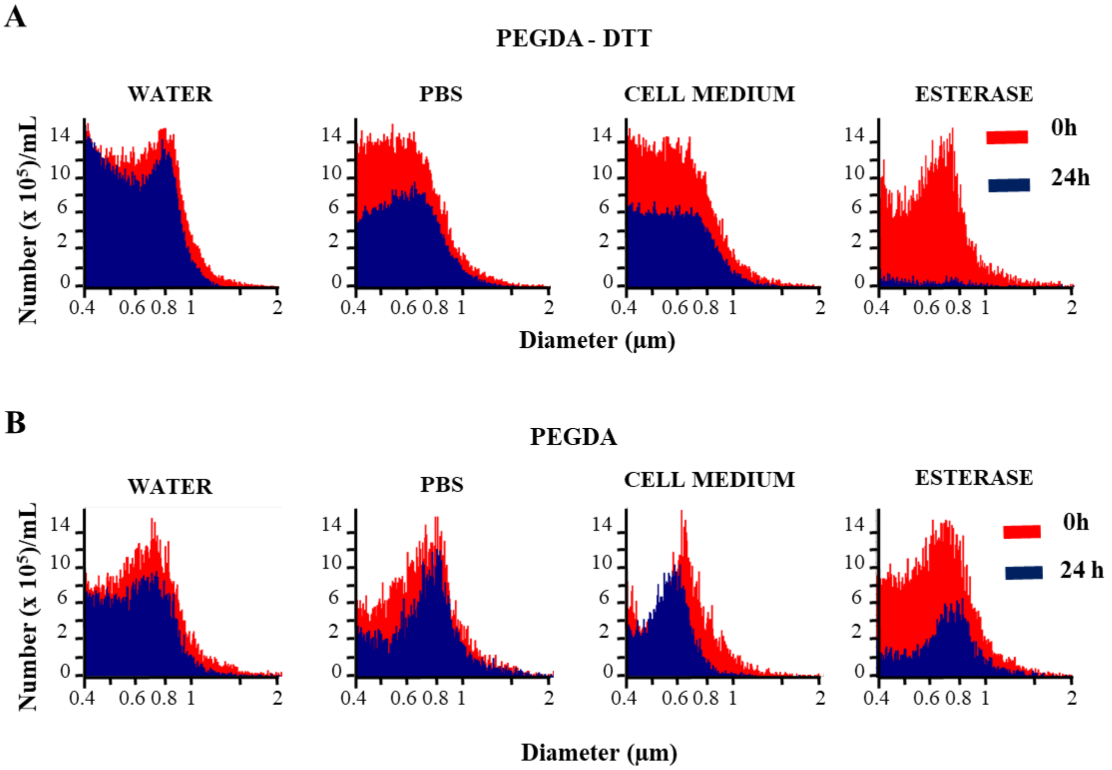

Figure S5. Alteration of µCGP morphology due to biodegradation. A. PEGDA-DTT µCGP degradation overtime: multisizer spectra at time 0 and 24h post incubation under different conditions. **B.** PEGDA (no DTT) µCGP degradation overtime: multisizer spectra at time 0 and 24h post incubation under different conditions.

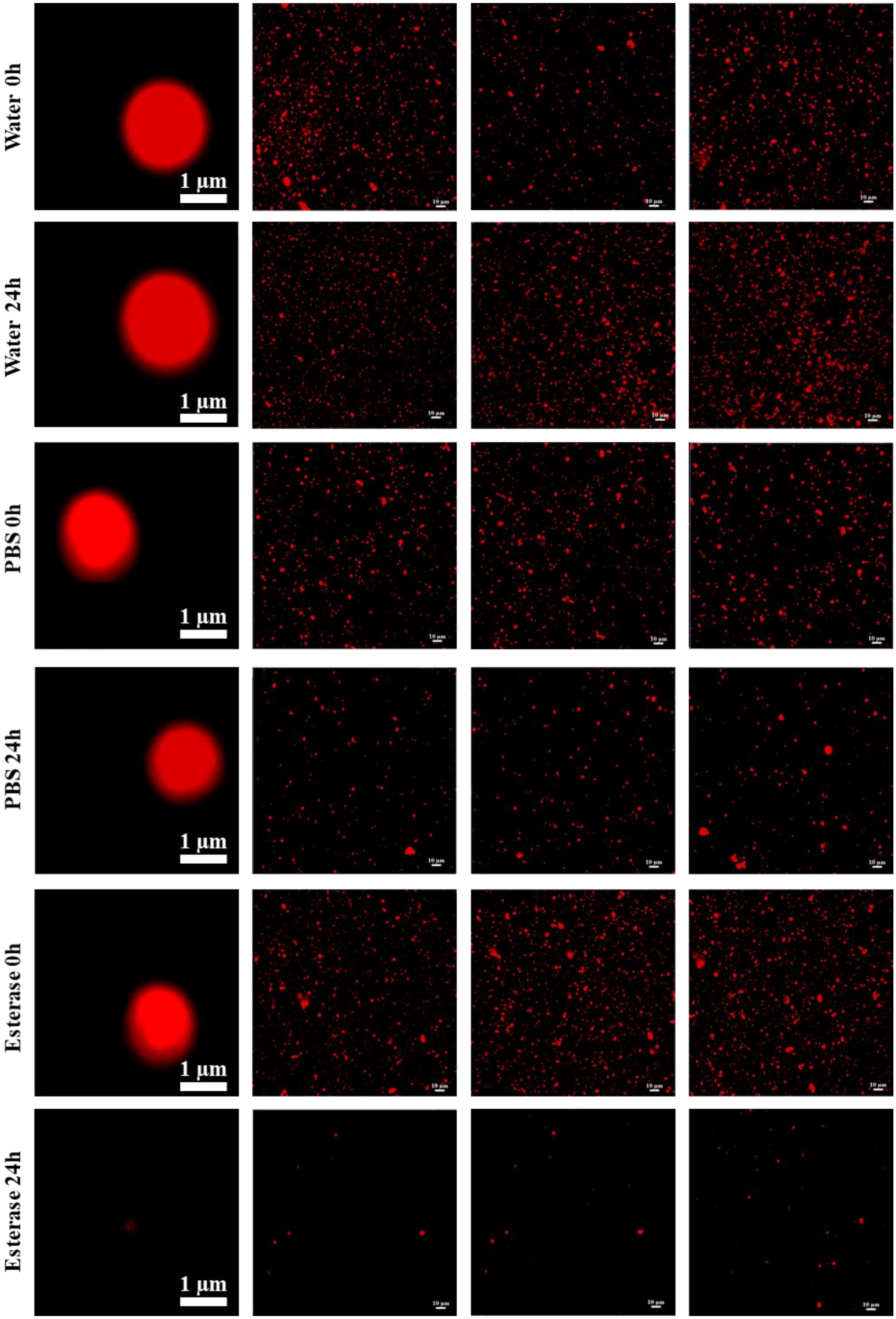
Water 0h
Water 24h
PBS 0h
PBS 24h
Esterase 0h
Esterase 24h
1 µm

Figure S6. Assessing the µCGP biodegradation via fluorescent microscopy. Confocal fluroescent images showing the progressive break down of µCGP under different conditions. The numerosity and fluorescent intensity decrease as the particle degrades. On the left higher magnification of the µCGP under different conditions and on the right different regions of interests imaged at a lower magnification scale.

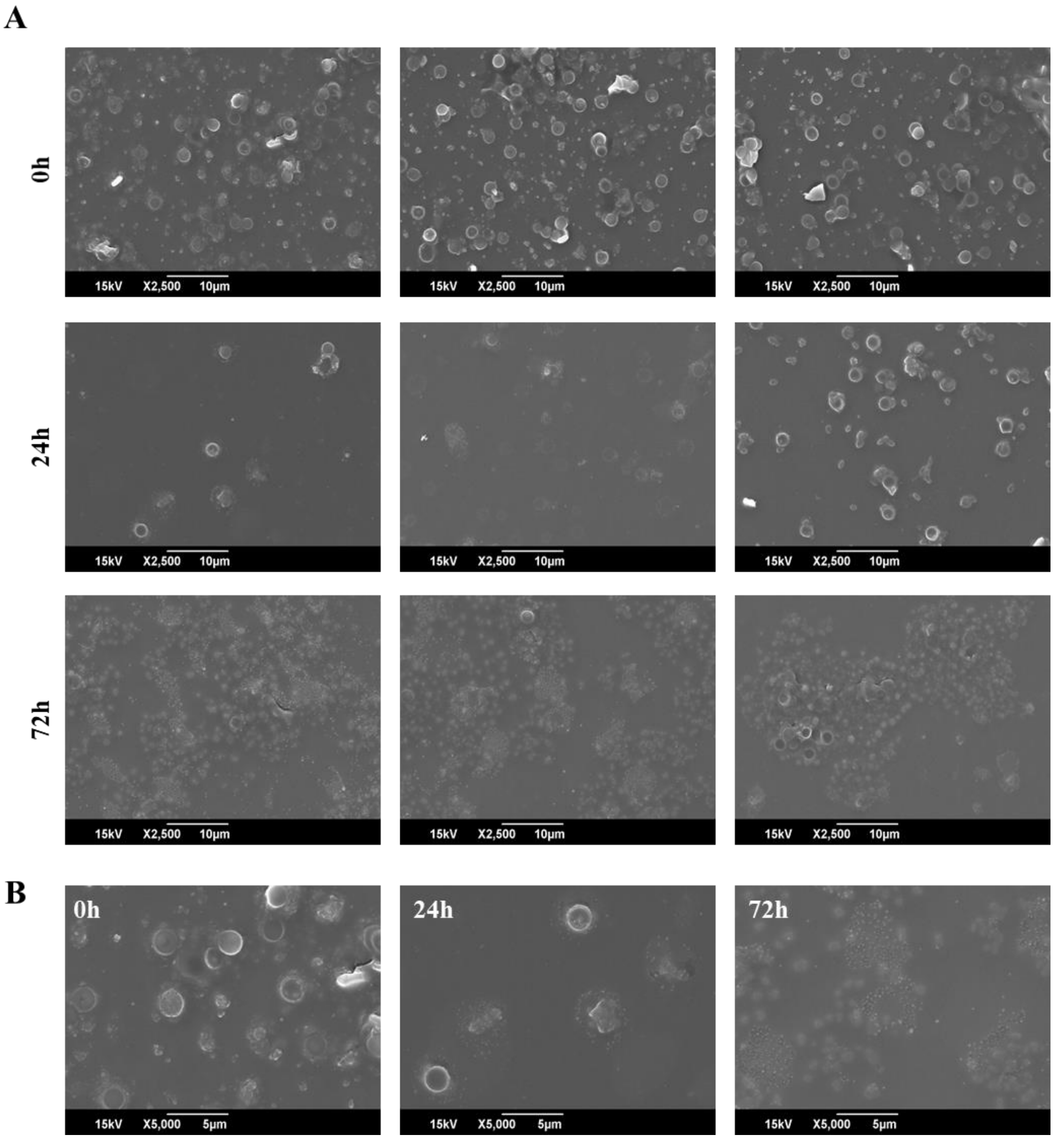

Figure S7. Assessing the µCGP biodegradation via SEM. A. SEM images showing the µCGP degradation in esterase solution after 0, 24, and 72h. **B.** SEM images showing the µCGP degradation in esterase solution after 0, 24, and 72h at higher magnification. At 72h, the particles lost their native morphological features.

Drug loading and release studies. To boost the amount of drug per single µCGP, first the loading of DTXL in the spherical polymeric nanoconstructs was optimized. In particular, different input amounts of DTXL were considered to prepare the DTXL-SPN. As shown in **Figure S8A**, the increase in DTXL input did not affect the SPN physico-chemical properties returning a uniform and monodispersed size distribution at the DLS with an average size around 160 nm for all the different configurations. Conversely, the different input amounts of DTXL did affect the drug loading and the 3 mg input conditions were selected for the highest loading (600 µg). Lower and higher input amounts returned lower DTXL amounts (**Figure S8B**). The encapsulation efficiencies for the 2 and 3 mg inputs were similar, and around 25% (**Figure S8C**). Interestingly, DTXL-µCGP obtained via the 3 mg DTXL-SPN (600 µg) led to a 2.5-fold increase in total drug loading as compared to DTXL-µCGP obtained via 2 mg DTXL-SPN (400 µg). The drug loading for the DTXL-µCGP was about five times higher than that typically measured for discoidal polymeric nanoconstructs (DTXL-DPN) previously realized by the authors using PLGA and molecular DTXL (**Figure S8D)**.[2] The release of the DTXL-SPN from the µCGP was also studied at physiological pH and mild acidic conditions and the results are presented in **Figure S8E-F** and directly compared to the release profiles obtained from SPN. [2] The results confirmed that the SPN confinement in the PEG matrix of µCGP reduces significantly the DTXL release, especially within the early time points and that an acidic environment accelerate the drug release overtime.

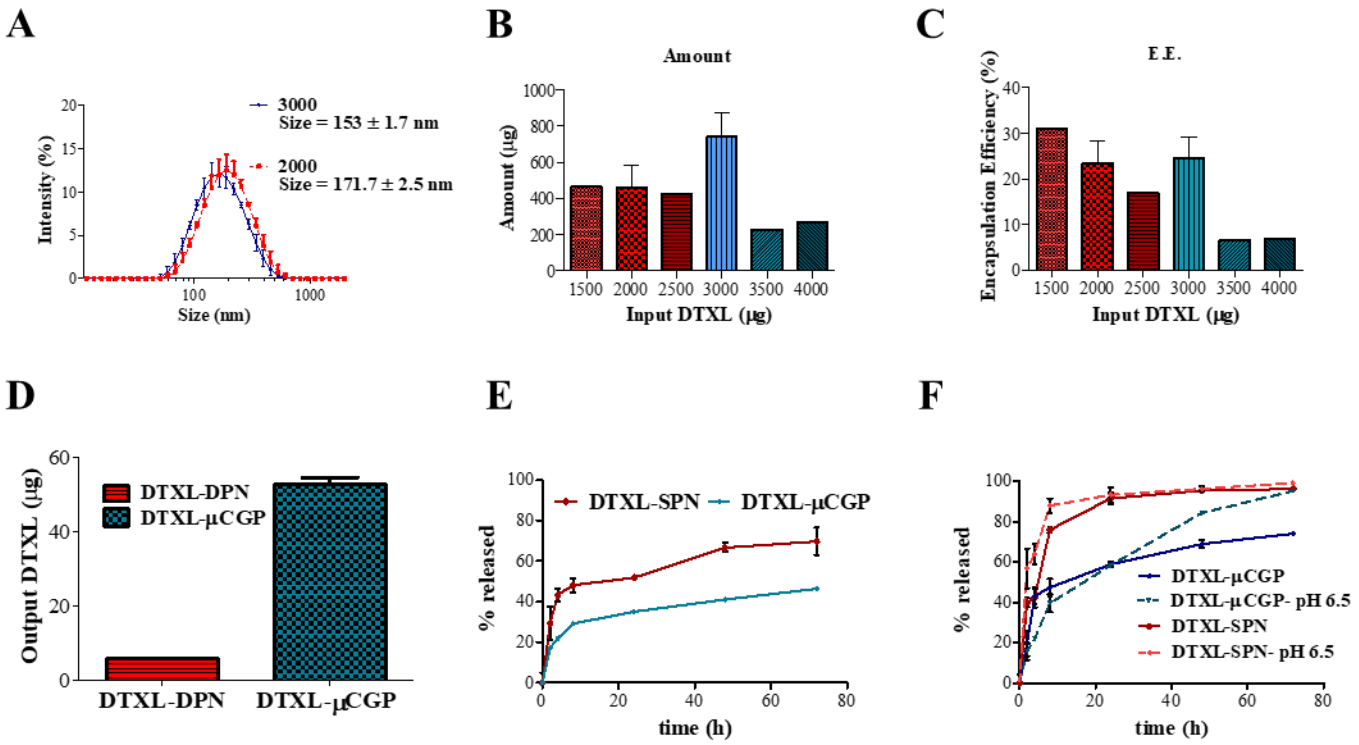

Figure S8. Loading and release of DTXL from µCGP. A. DLS analysis of DTXL-SPN made with 2,000 and 3,000 µg of DTXL input. Data are expressed as mean ± s.d., n = 3 independent samples per particle type; **B.** Drug loading into SPN varying the initial DTXL input. Data are expressed as mean ± s.d., n = 3 independent samples per particle type; **C**. Encapsulation efficiency in DTXL-SPN starting from different DTXL input. Data are expressed as mean ± s.d., n = 3 independent samples per particle type; **D**. DTXL loading into µCGP and DPN starting from the same DTXL input (600 µg DTXL input). Data are expressed as mean ± s.d., n = 3 independent samples per particle type; **E**. Drug release from DTXL-SPN and DTXL-µCGP in a confined volume (2 mL). Data are expressed as mean ± s.d., n = 3 independent samples per particle type; **F**. Drug release from DTXL-SPN and DTXL-µCGP under infinite sink conditions (4 L) at pH 7.4 and 6.5. Data are expressed as mean ± s.d., n = 3 independent samples per particle type.

µCGP internalization and in vitro cytotoxicity on breast cancer cells. The µCGP interaction with the triple negative breast cancer (TNBC) cells MDA-MB231 was studied via confocal microscopy by incubating cells with fluorescently labeled µCGP up to 96h. Results are reported in **Figure S9** and demonstrate that µCGP (red fluorescence) were not internalized but would rather deposit next to the cell body and progressively release the SPN (purple fluorescence) thereof. The interaction of µCGP with murine macrophages (RAW 264.7) was also studied using confocal fluorescent microscopy imaging and incubating particles and cells for up to 24h. Results are presented in **Figure S10** demonstrating that µCGP (red fluorescence) are not readily uptaken by professional phagocytic cells and stay attached to the cell membrane, likely due to their shape and flexibility, as previously documented by the authors and others[3, 4].

The in vitro cytotoxic potential of DTXL -µCGP was assessed via a MTT assays, for up to 96h of incubation with MDA-MB-231 cells. The results depicted in **Figure S11** show an overall lower cytotoxic activity of the µCGP as compared to the DTXL-SPN alone or the free DTXL. This indeed must be ascribed to the hierarchical architecture of the DTXL-µCGP and different kinetics of DTXL release and intracellular distribution. Finally, MTT with empty µCGP were performed to confirm the absence of any toxic effect due to the platform per se (**Figure S12**)

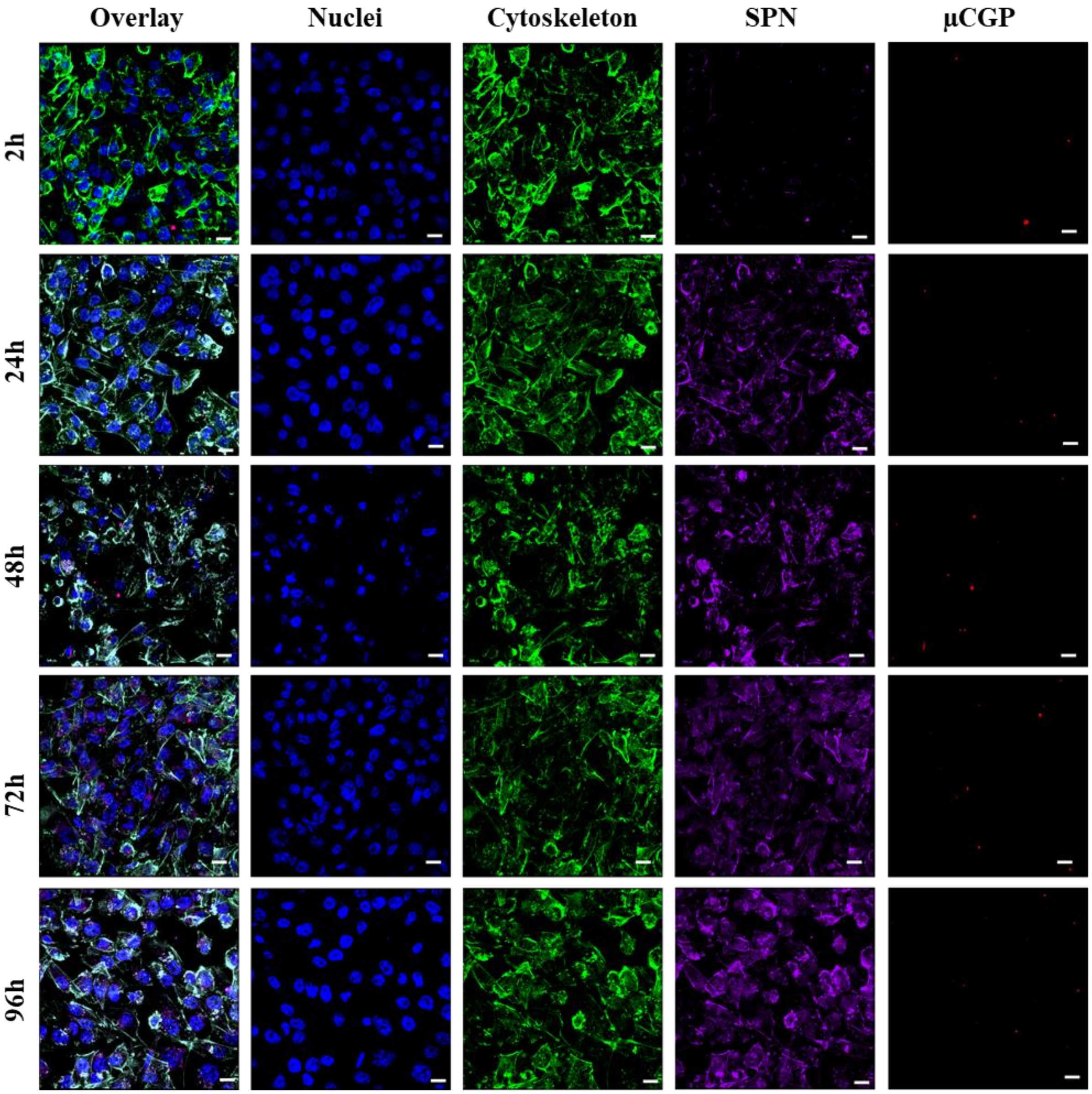

Figure S9. **Confocal microscopy images of MDA-MB 231 cells incubated with μCGP**. Cy5-labeled μCGP, loaded with RhB-SPN, were incubated with MDA-MB-231 for 2, 24, 48, 72 and 96h. Images show the progressive release of SPN (purple) from the μCGP matrix (red) and their consequent accumulation within the tumor cells (green: cell cytoskeleton; blue: cell nuclei). Scale bar: 10 μm.

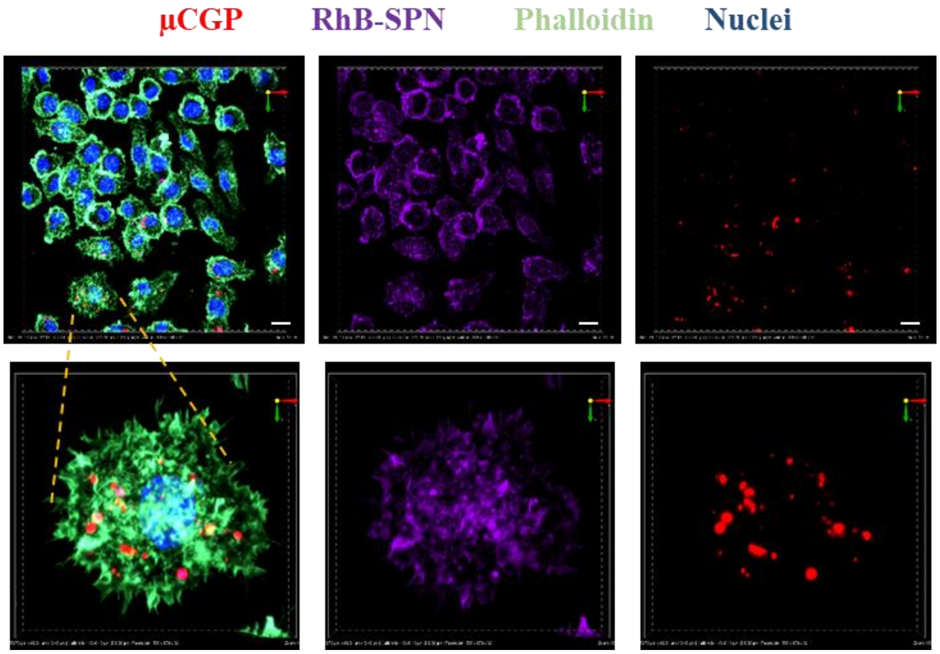

Figure S10. **Confocal microscopy images of RAW264.7 cells incubated with μCGP**. Confocal microscopy images of phagocytic RAW 264.7 cells incubated with μCGP, labeled with Cy5-PEG and loaded with RhB-SPN, for 24h. Images show μCGP adhering on the cell membrane of macrophages and the progressive release of SPN (red) and their consequent accumulation within the phagocytic cells (green: cell cytoskeleton; blue: cell nuclei). Scale bar: 10 μm.

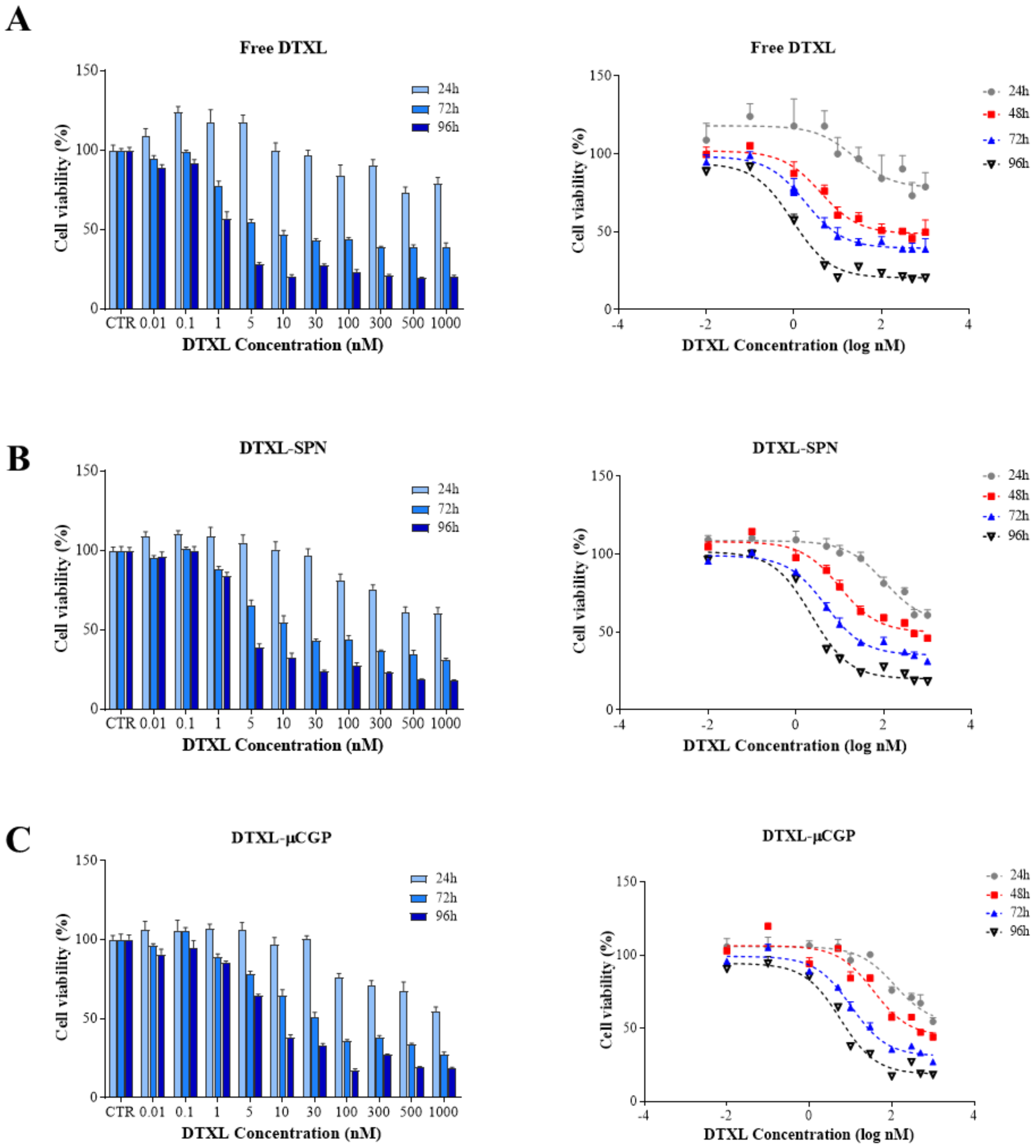

Figure S11. Cell viability studies on MDA-MB231 cells. Cytotoxicity profiles for free DTXL, DTXL-SPN, DTXL-μCGPs on triple negative breast cancer cells up to 96h. Data are expressed as mean ± s.d., n = 10 independent samples (R^2 values ≥ 0.9).

IC_{50} (nM)	24 h	48 h	72 h	96 h
Free DTXL	23.9	4.1	1.83	0.93
DTXL-SPN	102.5	10	4.75	2.27
DTXL-µCGP	118.3	36.3	10.3	5.6

Table S2. Tables provide the numerical values for IC_{50} up to 96 hrs.

LD_{50} nM	24 h	48 h	72 h	96 h
Free DTXL	-	100	8.13	1.38
DTXL-SPN	-	501.2	16.6	3.89
DTXL-µCGP	-	354.8	26.9	7.94

Table S3. Tables provide the numerical values for LD_{50} up to 96 hrs.

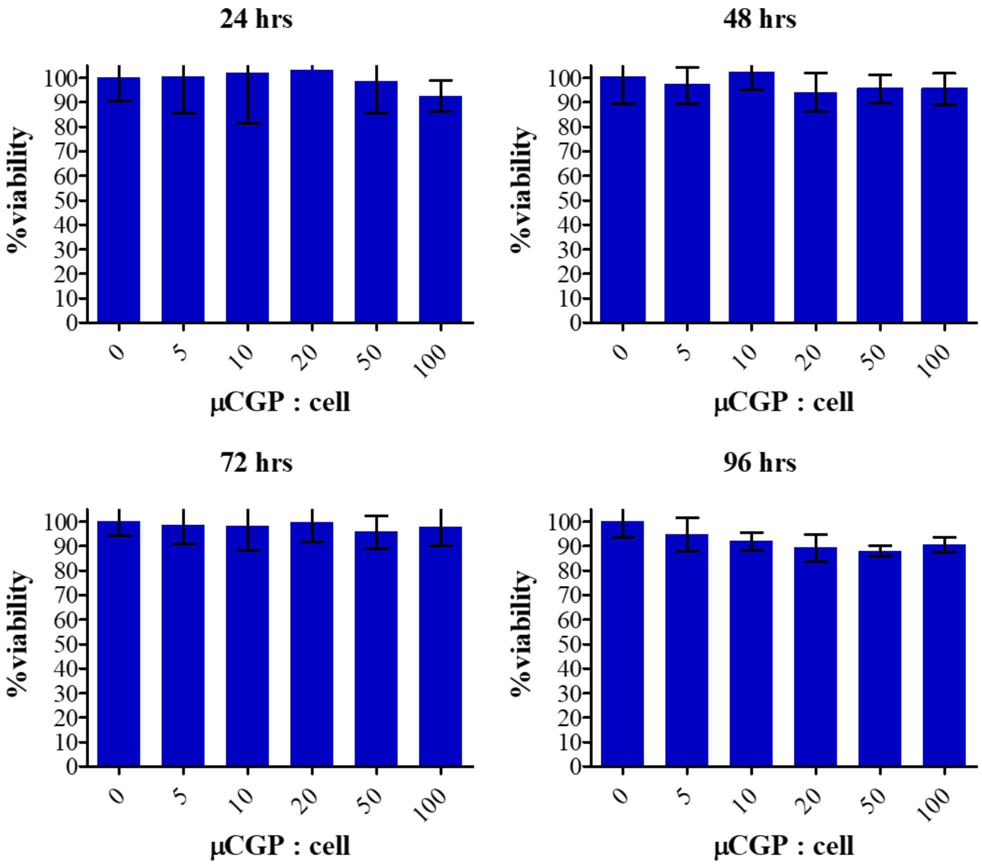

Figure S12. Cell viability studies on MDA-MB231 cells. Cytotoxicity profiles for empty μCGPs on triple negative breast cancer cells up to 96h. Data are expressed as mean ± s.d., n = 10 independent samples.

Preclinical imaging and therapeutic performance of DTXL-μCGP. An experimental murine model of TNBC-derived lung metastasis was established to demonstrate the therapeutic efficacy of DTXL-μCGP as compared to freely administered DTXL and DTXL-SPN. **Figures S13-16** collect the entire panel of the in vivo bioluminescence (BLI) images for all the treated mice, and for all the experimental group – intravenously injected free DTXL, DTXL-SPN, DTXL-μCGP, and saline injected control mice (CTR). While for all the experimental groups, the bioluminescent signal grew over time, mice treated with DTXL-μCGP experienced a decrease

and stabilization of the BLI signal demonstrating the therapeutic performance of the microgel-based drug delivery platform in arresting metastasis development.

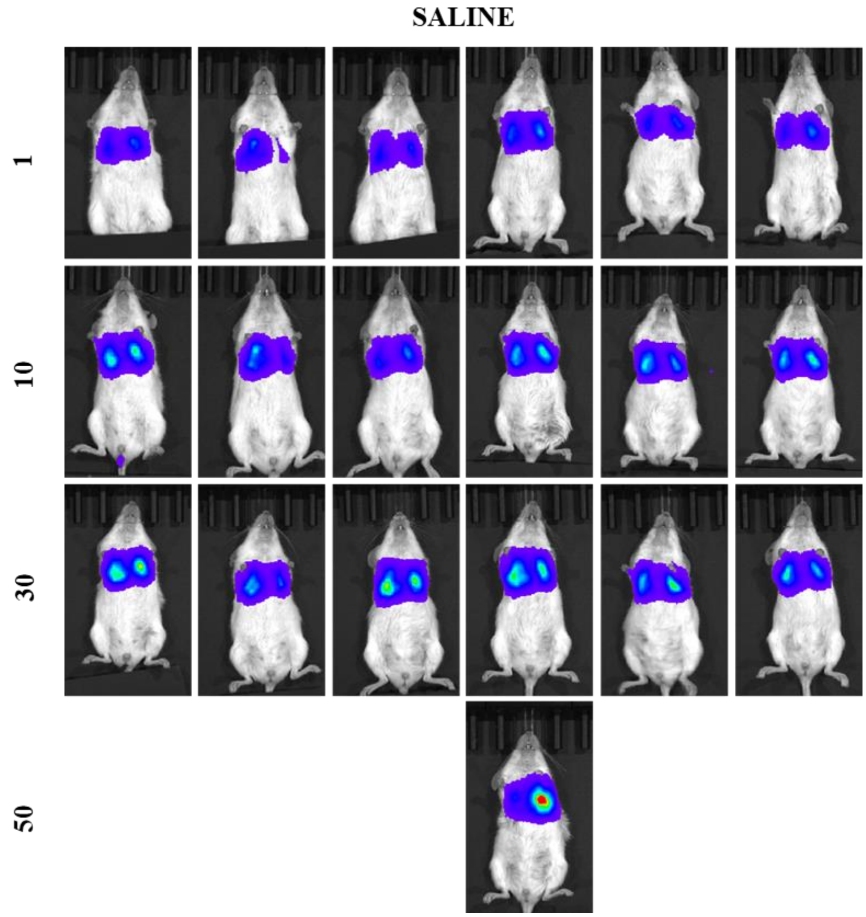

Figure S13. Bioluminescence analysis in TNBC-derived lung metastasis model – Saline control. Representative IVIS images taken at 1, 10, 30 and 50 days post treatment initiation for mice injected with saline showing the progressive growth of the metastatic nodules within the lung tissue.

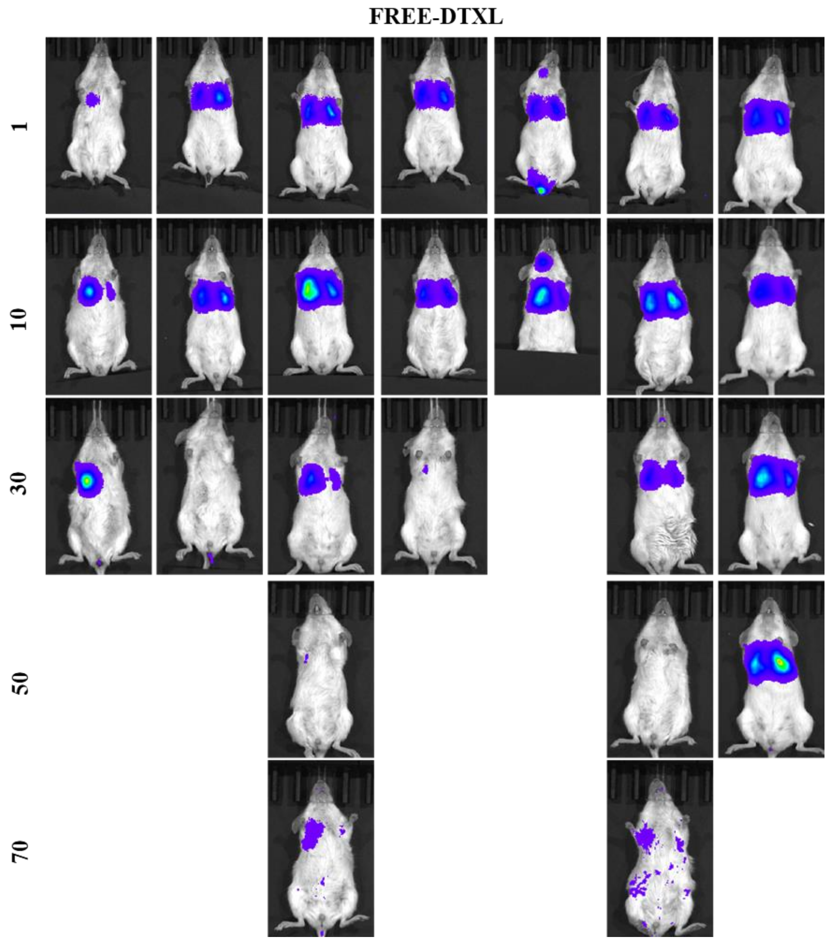

Figure S14. Bioluminescence analysis in TNBC-derived lung metastasis model – free DTXL. Representative IVIS images taken at 1, 10, 30, 50, and 70 days post treatment initiation for mice injected with free DTXL showing the progressive growth of the metastatic nodules within the lung tissue.

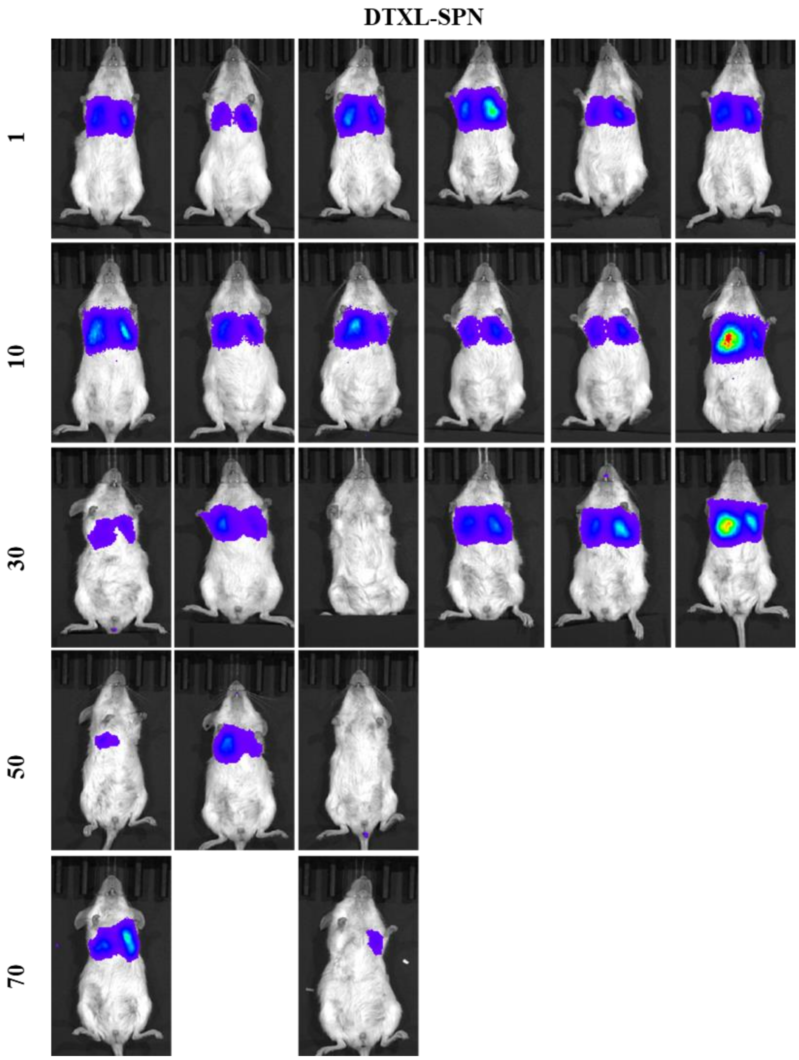

Figure S15. Bioluminescence analysis in TNBC-derived lung metastasis model – DTXL-SPN. Representative IVIS images taken at 1, 10, 30, 50, and 70 days post treatment initiation for mice injected with DTXL-SPN showing the progressive growth of the metastatic nodules within the lung tissue.

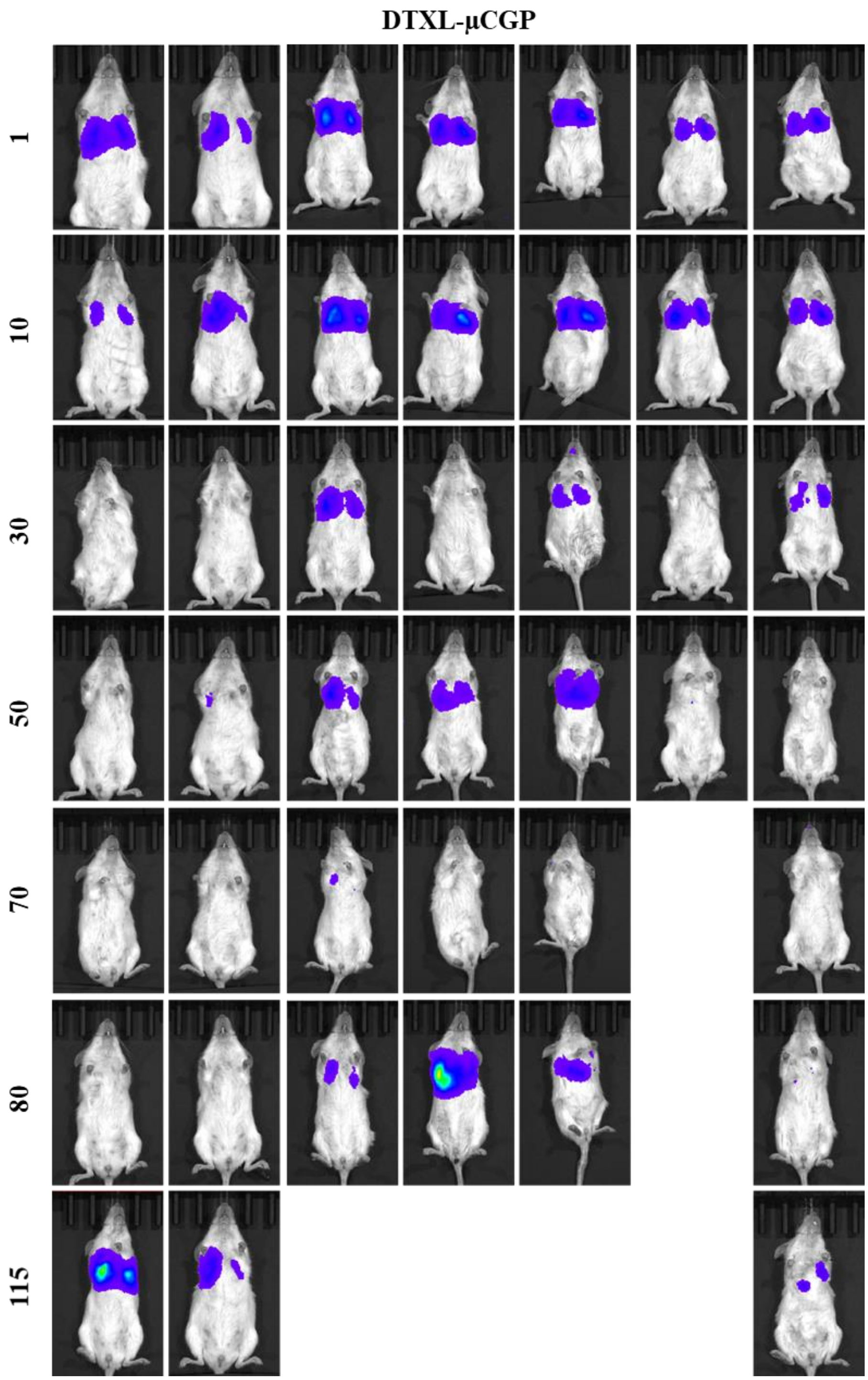

Figure S16. Bioluminescence analysis in TNBC-derived lung metastasis model – DTXL-μCGP. Representative IVIS images taken at 1, 10, 30, 50, 70, 80, and 115 days post treatment initiation for mice injected with DTXL-μCGP showing the control in growth of the metastatic nodules and much longer survival of the mice, as compared to the other 3 experimental groups.

Histological analysis of lung nodules. Standard procedures were followed to perform histological analysis on lung nodules of mice from each experimental group. The H&E staining in **Figure 6** of the main text shows the presence of multiple and large nodules (≥ 100 μm) in the control groups with respect to the mice treated with DTXL-μCGP. Also, the characteristic alveolar structure of the lung tissue is preserved only in the DTXL-μCGP group, while in all the other cases the uncontrolled growth of the metastatic nodes irreversibly alters the native tissue. Significant amounts of red blood cells are also observed in the control experimental groups, which is consistent with the progressive tissue damage induced by the malignancy progression. **Figure S17** presents photos of two types of lungs extracted from the mice. Both lungs bear nodules (yellow arrow) that are sufficiently large to be seen by eye. However, the tissue on the left has a regular and uniform coloration whereas the tissue on the right is characterized by a darker color possibly related to the presence of multiple hemorrhages.

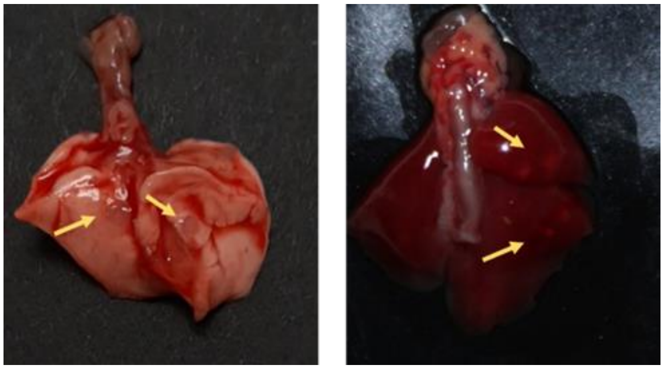

Figure S17. ex-vivo lung nodules identification. Representative photos showing lungs of a CTR (left) and Free DTXL treated mice with large metastasis (yellow arrow) and different tissue coloration. The right lungs appear darker, which might be indicative of extensive hemorrhaging processes.

Weight variation of the mice over the curse of the in vivo experiment. The weight of the treated mice was assessed periodically to identify any possible toxicity effect on animals. The variation in weight for all the mice is presented below in **Figure S18**. Most of the mice treated with DTXL-μCGP gained weight over time.

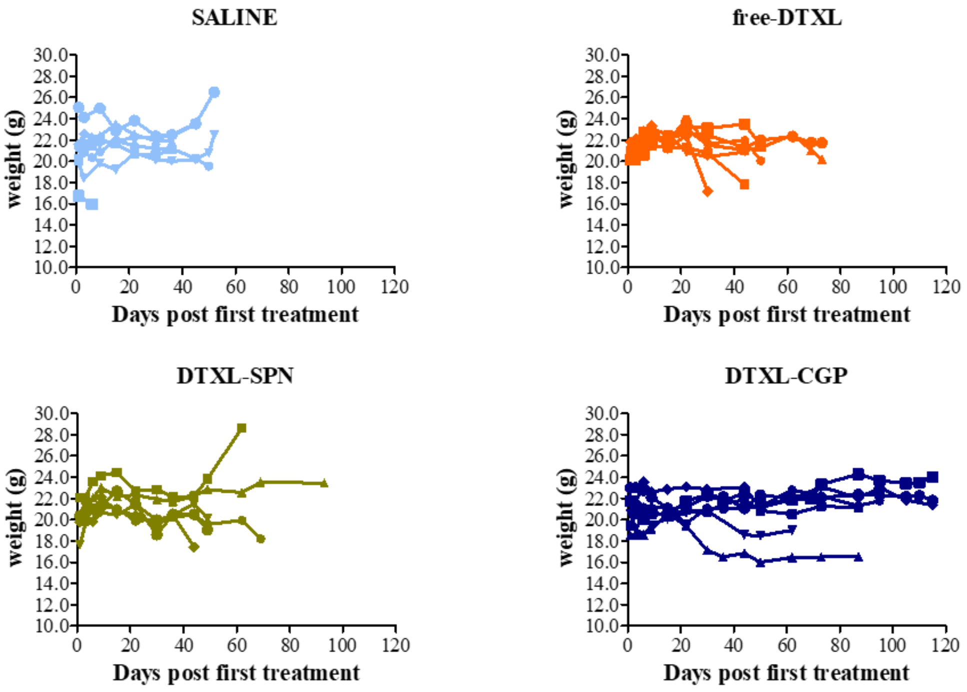

Figure S18. Mouse weight variation over time. Weight (g) of individual animals over the course of the in vivo experiments, for each experimental group (n ≥ 6).

Biodistribution analysis of μCGP and SPN. To demonstrate the preferential accumulation of μCGP into the lung nodules, four different techniques were employed, namely confocal microscopy, IVIS imaging, UPLC analyses, and nuclear imaging. First, confocal microscopy analyses were conducted on histological sections to identify fluorescently labeled μCGP and SPN. The μCGP were directly tagged with Cy5-PEG (red color) while RhB-SPN (purple) were dispersed within the hydrogel particle matrices. **Figure S19** documents the intra-tumor localization of μCGP at significant higher amounts as compared to directly injected SPN. Moreover, from these pictures it can be appreciated the mechanism of action of μCGP. Given their size, they are not expected to be internalized by cells. On the contrary, they accumulate at the vascular level and progressively release the smaller RhB-SPN, which, being much smaller, penetrate deeper and more uniformly into the tissue. Differently, intravenously injected RhB-SPN were seen to accumulate within the malignant mass only moderately. The different accumulation of intravenously administered μCGP and SPN is in agreement with the observed therapeutic response.

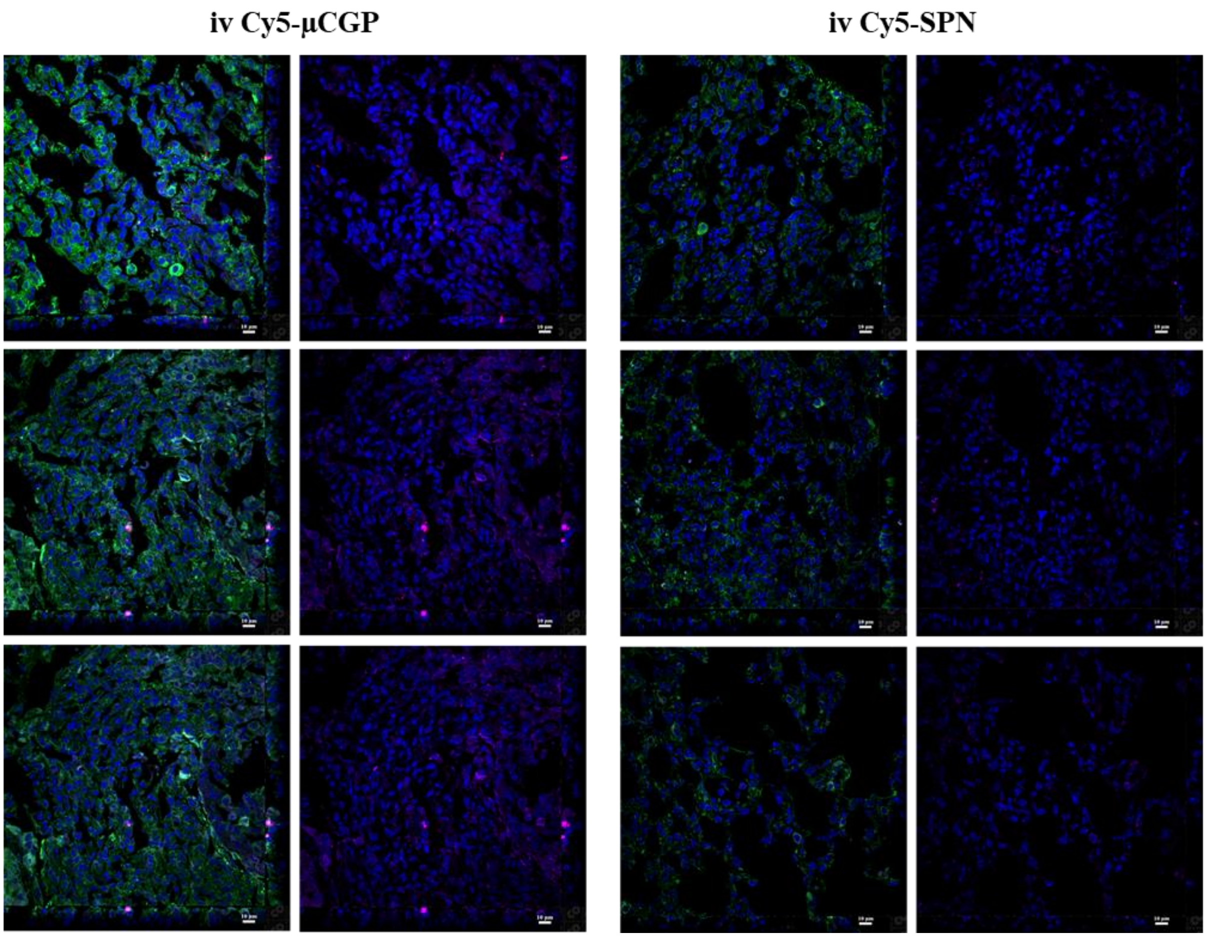

Figure S19. μCGP biodistribution studies - Confocal microscopy images of lung tissue slices. (left) Localization of intravenously injected Cy5-μCGP (red spot) and released RhB-SPN (purple) and (**right**) localization of intravenously injected Cy5-SPN in the lung metastatic nodules (green: aLUC staining) (scale bar = 10 μm).

Second, IVIS imaging was used to study the overall biodistribution of fluorescently labeled Cy5-μCGP and Cy5-SPN. A comparison was performed for the malignant tissue as well as other organs. *ex vivo* IVIS images of the main organs and lungs collected 24h post intravenous injection of Cy5-μCGP or Cy5-SPN (**Figure 6C** of the main text) report a significantly higher lung accumulation of the μCGP and their co-localization with the metastatic nodules. On the contrary, the accumulation of intravenously administered Cy5-SPN was minimal in the lungs. Importantly, these data demonstrate that the SPN biodistribution can be redirected upon their encapsulation within the hydrogel network of the μCGP.

Third, UPCL-MS/MS was used to quantify the amount of DTXL accumulating within the lungs and the major organs. The results are presented in **Figure 6** (main text) and expressed as the absolute percentage of the injected dose (%ID) and its value normalized by the mass of the tissue (%ID/g). In accordance with the biodistribution imaging studies and the *in vivo* therapeutic response, the amount of DTXL deposited within the lungs following a single DTXL-μCGP injection was about two times higher than that measured for free-DTXL and DTXL-SPN. As per the other organs, in general, a low amount of DTXL was recovered in the case of free-DTXL and DTXL-SPN. This could be explained by the rapid DTXL clearance mostly through the hepatobiliary circulation. Conversely, the total amount of DTXL recovered in mice injected with DTXL-μCGP was about 50% at the same time point, demonstrating the ability of this drug delivery platform to slowly release and preserve DTXL over time. **Figure S20** shows the direct comparison for all the analyzed organs and the different experimental groups with statistical analysis.

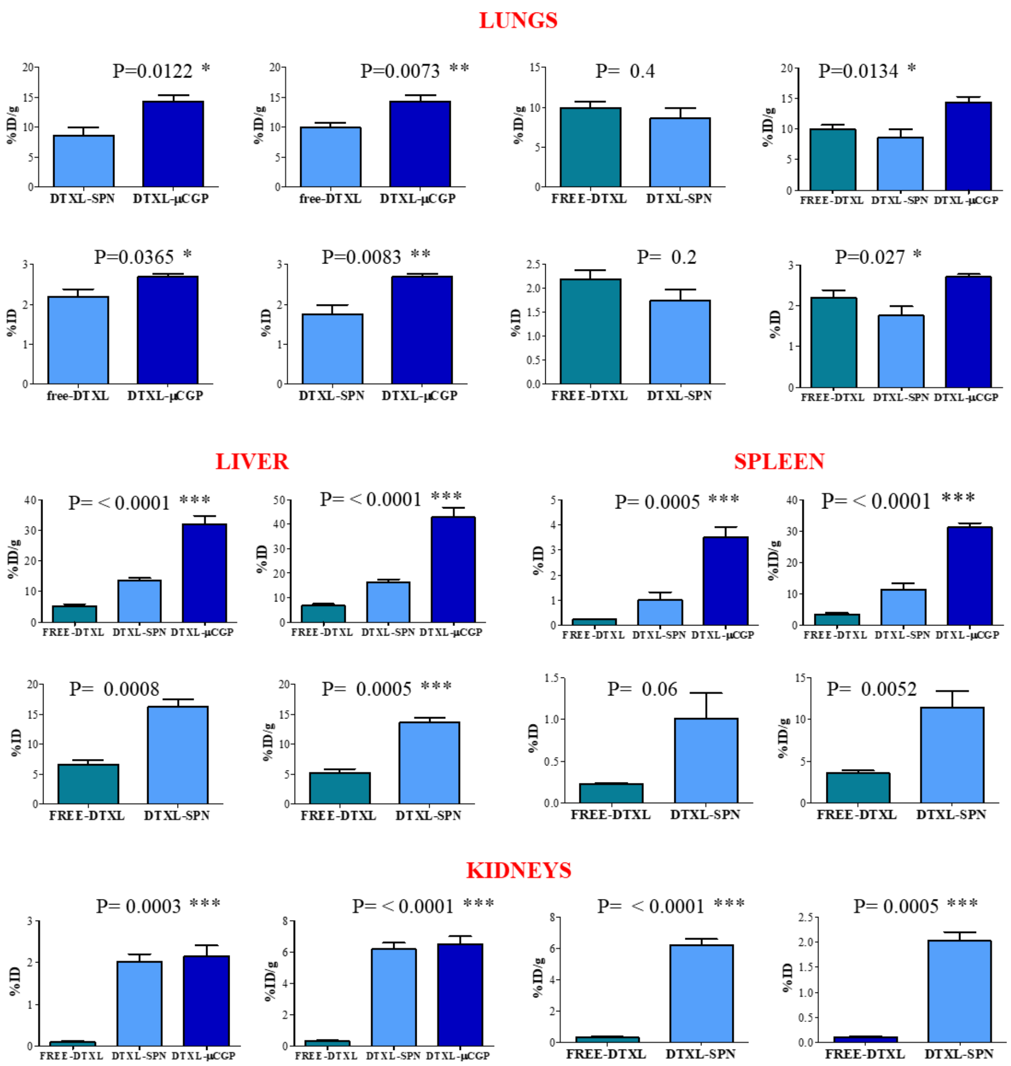

Figure S20: μCGP biodistribution studies – statistical analyses for DTXL intra tissue quantification. Statistics of the DTXL accumulation into the major organ expressed as %ID and %ID/g at 24h post systemic administration of free DTXL, DTXL-SPNs and DTXL-CGP, respectively. Data are presented as the average ± SD, n ≥4 mice per experimental group.

Finally, for the fourth method, the preferential μCGP accumulation in the lungs was observed via nuclear imaging. **Figure S21** documents positron emission tomography (PET) images of μCGP radiolabeled with ^{64}Cu accumulating within the TNBC-derived lung metastasis model. ^{64}Cu-μCGP were realized by including PEG-DOTA conjugates for the chelation of Cu^{2+}-radioactive ions with the DOTA cage. ^{64}Cu-μCGP (4.7 MBq) were administered in mice bearing lung metastasis and scanned at 1 and 24h post injection (p.i.). **Figure S21A** shows

^{64}Cu-μCGP maximum intensity projection (**left**) PET images at 1h p.i. with high lung uptake, also highlighted by the transverse slice (**right**). **Figure S21B** shows ^{64}Cu-μCGP maximum intensity projection (**left**) PET images at 24h p.i. with lung uptake; again highlighted in the transverse image (**right**). Indeed, given the inevitable accumulation of the μCGP in the liver, their progressive biodegradation and the high affinity of ^{64}Cu for the hepatocytes, the hypersensitivity in the hepatic and abdominal area was expected.

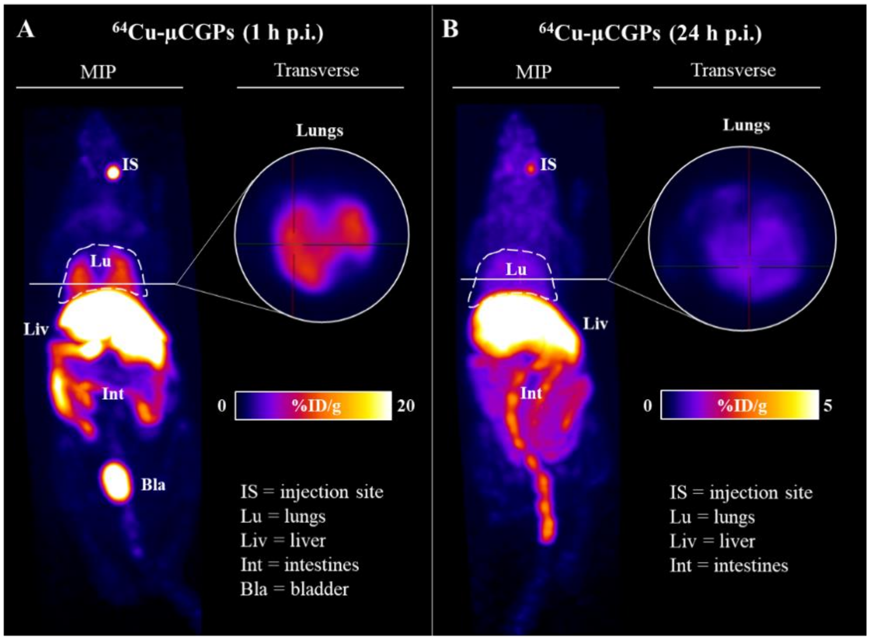

Figure S21. μCGP biodistribution studies – Nuclear imaging of ^{64}Cu-μCGP distribution. A. ^{64}Cu-μCGP maximum intensity projection (**left**) PET images 1 h p.i. showing high lung uptake as highlighted in the transverse slice (**right**). **B**. ^{64}Cu-μCGP maximum intensity projection (**left**) PET images 24 h p.i. also showing lung uptake as highlighted in the transverse image (**right**).

REFERENCE

1. Di Mascolo, D.; Basnett, P.; Palange, A. L.; Francardi, M.; Roy, I.; Decuzzi, P., Tuning core hydrophobicity of spherical polymeric nanoconstructs for docetaxel delivery. *Polymer International* **2016,** *65* (7), 741-746.
2. Felici, A.; Di Mascolo, D.; Ferreira, M.; Lauciello, S.; Bono, L.; Armirotti, A.; Pitchaimani, A.; Palange, A. L.; Decuzzi, P., Vascular-confined multi-passage discoidal

nanoconstructs for the low-dose docetaxel inhibition of triple-negative breast cancer growth. *Nano Research* **2022,** *15* (1), 482-491.

3. Key, J.; Palange, A. L.; Gentile, F.; Aryal, S.; Stigliano, C.; Di Mascolo, D.; De Rosa, E.; Cho, M.; Lee, Y.; Singh, J., Soft discoidal polymeric nanoconstructs resist macrophage uptake and enhance vascular targeting in tumors. *ACS nano* **2015,** *9* (12), 11628-11641.

4. Palomba, R.; Palange, A. L.; Rizzuti, I. F.; Ferreira, M.; Cervadoro, A.; Barbato, M. G.; Canale, C.; Decuzzi, P., Modulating phagocytic cell sequestration by tailoring nanoconstruct softness. *Acs Nano* **2018,** *12* (2), 1433-1444.
